# Renoprotective effects of human L-type fatty acid-binding protein (hL-FABP) in rhabdomyolysis-induced acute kidney injury

**DOI:** 10.1038/s41598-025-34045-9

**Published:** 2025-12-26

**Authors:** Kazuho Inoue, Seiko Hoshino, Keiichi Ohata, Takeshi Sugaya, Kenjiro Kimura, Yugo Shibagaki, Atsuko Kamijo-Ikemori

**Affiliations:** 1 Department of Anatomy, St. Marianna University School of Medicine, Kawasaki, Kanagawa Japan; 2 Division of Nephrology and Hypertension, Department of Internal Medicine, St. Marianna University School of Medicine, Kawasaki, Kanagawa Japan; 3 JCHO Tokyo Takanawa Hospital, Tokyo, Japan; 4 Institute for Animal Experimentation, St. Marianna University Graduate School of Medicine, Kawasaki, Kanagawa Japan

**Keywords:** L-type fatty acid-binding protein (L-FABP), Rhabdomyolysis, Acute kidney injury, Lipid peroxidation, Ferroptosis, Acute kidney injury, Experimental models of disease

## Abstract

L-type fatty acid-binding protein (L-FABP) in proximal tubules is reported to reduce oxidative stress. This study investigated whether renal L-FABP mitigated rhabdomyolysis (RM)-induced acute kidney injury (AKI). Wild-type (WT) mice, which lack renal L-FABP expression, and human L-FABP (hL-FABP) chromosomal transgenic (Tg) mice, which express hL-FABP in proximal tubules, were divided into RM (WT-RM, Tg-RM) and control (WT-Cont, Tg-Cont) groups. RM was induced via intramuscular injection of 50% glycerol/PBS into the quadriceps, whereas controls received PBS alone. On day 1, the WT-RM and Tg-RM groups exhibited similar increases in serum myoglobin, renal dysfunction, heme oxygenase-1 expression, and tubulointerstitial damage. By day 3, the Tg-RM group demonstrated significant improvements in renal function, attenuation of tubulointerstitial damage, and decrease in macrophage infiltration compared with the WT-RM group. Lipid peroxidation marker 4-hydroxynonenal increased similarly in both RM groups on day 1, but its further elevation on day 3 was significantly suppressed in Tg-RM mice. Solute carrier family 7 member 11 on day 1 and glutathione peroxidase 4 on day 3 were significantly higher in Tg-RM mice than in WT-RM mice, which led to the suppression of ferroptosis. In conclusion, renal hL-FABP may prevent progression of RM-induced AKI through ferroptosis inhibition by antioxidative effects.

**Supplementary Information:**

The online version contains supplementary material available at 10.1038/s41598-025-34045-9.

## Introduction

Rhabdomyolysis (RM) is a condition that can be triggered by various factors, including trauma, intense exercise, drug intoxication, and infection^1^. It is characterized by the breakdown of skeletal muscle fibers, which leads to the release of intramuscular components into the systemic circulation. This can subsequently result in acute kidney injury (AKI). RM-induced AKI is associated with higher mortality than RM without AKI, and severe cases often require chronic renal replacement therapy. However, effective treatment options for RM-induced AKI remain limited. Therefore, identifying new therapeutic targets is essential for preventing its onset and progression^2^.

The primary pathogenic factors in RM-induced AKI are myoglobin, free heme, and other damage-associated molecular patterns released from damaged skeletal muscle. These components are filtered through the glomeruli and reabsorbed into proximal tubules, where they trigger oxidative stress and inflammation, leading to tubular cell death. Both myoglobin and free heme contain iron, which accumulates in the proximal tubules and induces iron-dependent oxidative stress. Recently, ferroptosis, a regulated form of cell death driven by iron-dependent lipid peroxidation, has been increasingly recognized for its role in RM-induced AKI^3^. Therefore, a molecule with antioxidative properties could be a potential therapeutic target for mitigating RM-induced AKI by inhibiting ferroptosis.

L-type fatty acid-binding protein (L-FABP) is a cytoplasmic protein that plays a key role in the intracellular transport of fatty acids and other lipophilic substances^4,5^ and is expressed in human proximal tubules. In kidney diseases induced by ischemic reperfusion, cisplatin, aristolochic acid, unilateral ureteral obstruction, angiotensin II, and aldosterone, human L-FABP (hL-FABP) has been reported to exert renoprotective effects by reducing oxidative stress and lipid peroxidation products in hL-FABP chromosomal transgenic (Tg) mice, as wild-type (WT) mice do not express L-FABP in their kidneys^6–11^. However, the role of renal hL-FABP in RM-induced AKI remains unclear. Therefore, this study investigated whether hL-FABP provides renoprotective effects against RM-induced AKI by reducing lipid peroxidation and inhibiting ferroptosis.

## Materials and methods

### Animals

This study adhered to the ARRIVE guidelines. All animal experiments were conducted in accordance with the ethical standards of St. Marianna University School of Medicine. The experimental protocol was approved by Animal Experiment Committee at the Institute for Animal Experimentation, St. Marianna University Graduate School of Medicine (Approval Nos. TG230526-2 and TG240528-3).

Male WT C57BL/6 mice were obtained from Japan SLC (Shizuoka, Japan). Since WT mice do not express L-FABP in their kidneys, male hL-FABP Tg mice derived from the C57BL/6 strain (patent no. WO0073791) were used^5,9^. In brief, a genomic DNA fragment containing the entire human L-FABP gene, including its 13-kb promoter region, was microinjected into fertilized eggs derived from C57BL/6 and CBA mice. ICR mice were used as recipients for the transfected eggs. The resultant transgenic mice were subsequently backcrossed with C57BL/6 mice for over ten generations to establish a congenic line. The male hL-FABP Tg mice used in this study were kindly provided by one of the coauthors, Prof. T. Sugaya (Time Well Medical Co., Ltd., Tokyo, Japan). The animals were maintained under specific pathogen-free conditions at a laboratory animal supplier (Japan SLC, Inc.). These male Tg mice expressed hL-FABP in their proximal tubules. All mice were housed at the Institute for Animal Experimentation, St. Marianna University School of Medicine, under controlled conditions (24 °C, 12-h light/dark cycle) with free access to standard laboratory chow (CE-2; CLEA Japan, Inc., Tokyo, Japan) and water.

### RM-induced AKI model

**Fig. 1:**
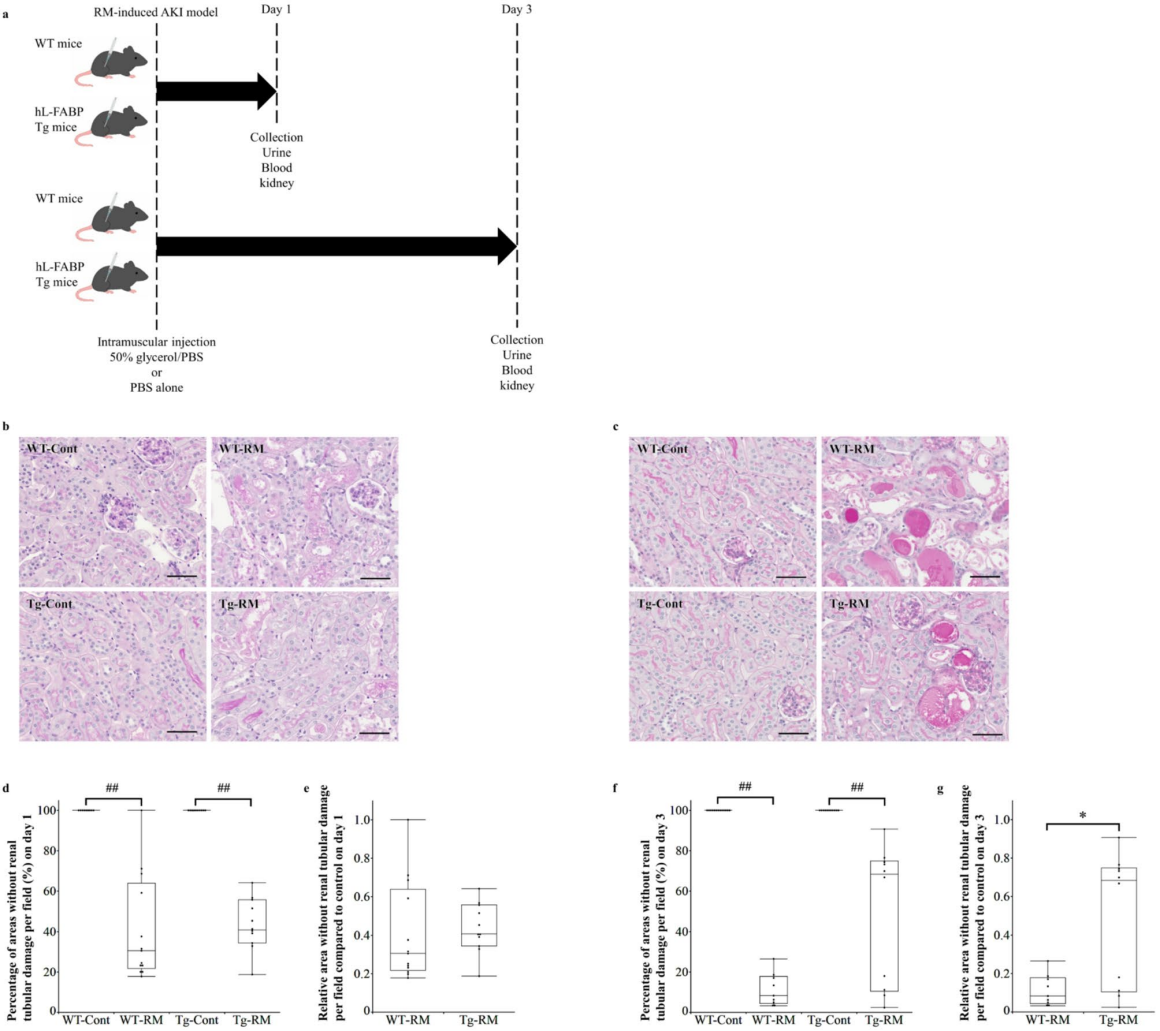
Experimental protocol and assessment of tubular damage by PAS staining in kidney sections. (**a**) Schematic illustration of the rhabdomyolysis (RM)-induced acute kidney injury (AKI) model. Male wild-type (WT) and human L-type fatty acid-binding protein chromosomal transgenic (hL-FABP Tg) mice were randomly assigned to either the RM or control (Cont) group. RM was induced by intramuscular injection of 50% glycerol/PBS, while control mice received PBS alone. On days 1 and 3, mice were sacrificed for collection of urine, blood, and kidney samples. (**b**,**d**,**e**) Representative images of each group on day 1 (**b**), percentage of areas without renal tubular damage per field (**d**), and comparison of relative area without renal tubular damage per field in the two RM groups respect to control (**e**). (**c**,**f**,**g**) Representative images of each group on day 3 (**c**), percentage of areas without renal tubular damage per field (f), and comparison of relative area without renal tubular damage per field in the two RM groups respect to control (**g**). Original magnification: x200. Scale bars = 50 μm. Comparisons among all groups were performed using the Kruskal–Wallis test followed by the Steel–Dwass post hoc test. Differences between the Tg-RM and WT-RM groups (normalized to each corresponding control group) were analyzed using the Mann–Whitney U test when Kruskal–Wallis test showed an overall group significance. All data are presented as medians with ranges. ^##^*p* < 0.01 vs. the corresponding group control; *p < 0.05 vs. WT-RM.

Male WT and Tg mice (8–12 weeks old, body weight 23–26 g) were assigned to either the RM group or control group (Fig. 1a). The RM model was established as previously described^12^. Mice in the RM group received an intramuscular injection of 50% glycerol/PBS at a dose of 7.5 mL/kg body weight, with half administered into the right quadriceps and the other half into the left quadriceps under 2% isoflurane inhalation anesthesia. The control group received an intramuscular injection of PBS alone at the same dose under 2% isoflurane inhalation anesthesia. At the end of the experiment, mice were euthanized by intraperitoneal injection of an overdose of pentobarbital sodium (400–500 mg/kg) for blood and kidney sample collection on days 1 and 3 (day 1, *n* = 13 for WT-RM, *n* = 11 for Tg-RM, *n* = 9 for WT-Cont, *n* = 10 for Tg-Cont; day 3, *n* = 9 for WT-RM, *n* = 10 for Tg-RM, *n* = 13 for WT-Cont, *n* = 11 for Tg-Cont). For urine collection, mice were individually housed overnight in metabolic cages with free access to tap water the day before euthanasia, and urine samples were collected immediately before euthanasia on days 1 and 3 (Fig. 1a).

### Serum and urinary biochemistry

In both WT and Tg mice, serum cystatin C and myoglobin levels as well as urinary mouse kidney injury molecule-1 (KIM-1) were measured using the Cystatin C Mouse ELISA Kit (BioVendor, Brno, Czech Republic), Mouse Myoglobin ELISA Kit (Abcam, Cambridge, UK), and Mouse TIM-1/KIM-1/HAVCR Quantikine ELISA Kit (R&D Systems, Inc., Minneapolis, MN), respectively. In Tg mice, urinary hL-FABP levels were measured using the Human L-FABP ELISA Kit (CMIC Holdings, Tokyo, Japan) and calculated as the daily excretion.

### Renal histological and Immunobiological analyses

Kidneys were fixed in 10% buffered formalin or methyl Carnoy’s solution, embedded in paraffin, and sectioned at a thickness of 3 μm. These sections were used for renal histological assessment, including periodic acid–Schiff (PAS) staining and immunohistochemistry. In PAS-stained specimens, areas without renal tubular damage—defined as tubular dilatation with epithelial atrophy, detachment of tubular epithelial cells from the basement membrane, loss of brush border or tubular cast formation—were measured in 10 nonoverlapping fields with x100 magnification, as severe and extensive tubular damage was observed in the WT-RM group on day 3. For immunostaining, a primary antibody against F4/80 (rat monoclonal antibody; 1:200, Cl: A3-1; Bio-Rad, Hercules, CA, USA) was used to identify macrophages. A secondary antibody, anti-rat IgG (Vector Laboratories, Burlingame, CA, USA), was used for detection. The signals were visualized using 3,3’-diaminobenzidine (DAB). To confirm the localization of myoglobin and hL-FABP in Tg mice, fluorescent immunostaining was performed using primary antibodies against myoglobin (rabbit monoclonal antibody; 1:500; ab77232; Abcam) and hL-FABP (mouse monoclonal antibody; 1:1000; CMIC Holdings). The M.O.M. Mouse Ig Blocking Reagent (Vector Laboratories) was used to minimize non-specific signals from the secondary antibody in hL-FABP staining. For detection, Alexa Fluor Plus 594-conjugated Anti-Rabbit IgG (1:500; Thermo Fisher Scientific, Waltham, MA, USA) and Alexa Fluor Plus 488-conjugated Anti-Mouse IgG (1:500; Thermo Fisher Scientific) were used. All renal histological and immunohistological analyses were performed using WinROOF (Mitani Corporation, Tokyo, Japan).

### Real-time polymerase chain reaction (PCR)

Total RNA was extracted from kidney tissues using the RNeasy Mini Kit (Qiagen, Valencia, CA, USA) and subsequently reverse-transcribed into cDNA using the PrimeScript™ RT Reagent Kit (Perfect Real Time) (Takara Bio Inc., Shiga, Japan). The mRNA expression levels of heme oxygenase-1 (HO-1, Mm00516005_m1) and 18 S ribosomal RNA (18 S, Mm03928990) were measured using TaqMan real-time PCR with StepOnePlus™ Real-Time PCR System (Applied Biosystems, Waltham, MA, USA). Target mRNA expression levels were normalized to 18 S expression.

### Assessment of proinflammatory chemokine

Frozen kidney samples were homogenized in T-PER Tissue Protein Extraction Reagent (Thermo Fisher Scientific) containing protease and phosphatase inhibitors. The homogenates were centrifuged at 15,000 rpm for 10 min to remove tissue debris, and the supernatants were collected. Protein concentrations were determined using the Bradford assay (Bio-Rad). Monocyte chemotactic protein-1 (MCP-1) levels were measured using the Mouse CCL2/JE/MCP-1 Quantikine ELISA Kit (R&D Systems).

### Western blotting

**Fig. 2:**
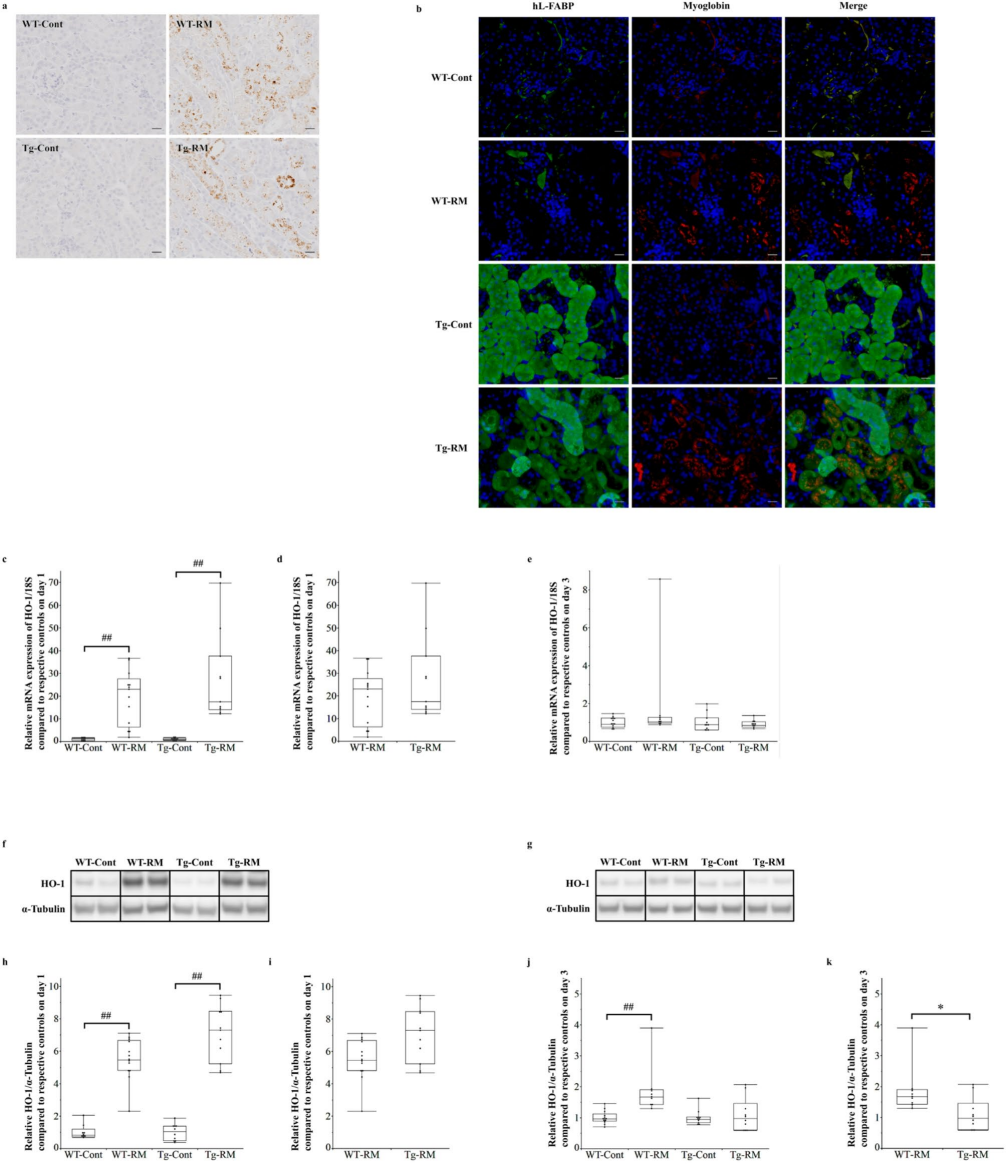
Immunohistochemistry for myoglobin, immunofluorescent staining for myoglobin and hL-FABP, and analysis of HO-1 expression. (**a**) Representative immunohistochemical staining images for myoglobin in kidney sections from each group on day 1. (**b**) Immunofluorescent staining for hL-FABP (green) and myoglobin (red) in kidney sections from each group on day 1. Nuclei were stained with DAPI (blue). Original magnification: x400 for all images. Scale bars = 20 μm. (**c**–**e**) Relative HO-1 mRNA expression normalized to 18 S rRNA on day 1 (**c**,**d**) and 3 (**e**), relative to respective control groups. (**f**,**g**) Western blot analysis of HO-1 in kidney tissues from each group on day 1 (f) and 3 (g). α-Tubulin was used as a loading control. Samples from the same experiment were processed in parallel for SDS polyacrylamide gel electrophoresis (SDS-PAGE) and Western blotting using different gels and membranes, and the image data obtained were cropped. Entire Western blot images are shown in Supplementary Figure S1. (**h**–**k**) HO-1 quantification normalized to α-Tubulin on day 1 (h, i) and 3 (j, k), relative to the respective control groups. Comparisons among all groups were performed using the Kruskal–Wallis test followed by the Steel–Dwass post hoc test. Differences between Tg-RM and WT-RM groups (normalized to each corresponding control group) were analyzed using the Mann–Whitney U test when Kruskal–Wallis test showed an overall group significance. All data are presented as medians with ranges. ^##^*p* < 0.01 vs. the corresponding group control; *p < 0.05 vs. WT-RM.

Proteins (15 µg per well) were separated using NuPAGE 4%–12% Bis-Tris gels (Thermo Fisher Scientific) and transferred onto a polyvinylidene fluoride membrane using HorizeBLOT 4 M and EzFastBlot HMW (ATTO, Tokyo, Japan). Before incubation with primary antibodies, the membranes were cut near the molecular weight of each target protein. The following primary antibodies were used: HO-1 (1:1000; #82206; Cell Signaling Technology, Danvers, MA, USA), phospho-nuclear factor kappa-B p65 (Ser536) (p-NF-κB, 1:1000; #3033; Cell Signaling Technology), NF-κB p65 (1:1000; #8242; Cell Signaling Technology), peroxisone proliferator-activated receptor α (PPARα, 1 µg/mL; ab126285; Abcam), inhibitor of κBα (IκBα, 1:1000; #4812; Cell Signaling Technology), 4-hydroxynonenal (4HNE, 15 µg/mL; MHN-020P; Japan Institute for the Control of Aging, NIKKEN SEIL Co., Ltd., Shizuoka, Japan), acyl-CoA synthetase long-chain family member 4 (ACSL4, 1:10000; ab155282; Abcam), cyclooxygenase-2 (COX-2, 1:1000; #12282; Cell Signaling Technology), solute carrier family 7 member 11 (SLC7A11, 1:1000; ab307601; Abcam), glutathione peroxidase 4 (GPX4, 1:1000; #52455; Cell Signaling Technology) and α-tubulin (1:8000; ab176560; Abcam). Secondary antibodies were applied as follows: Rabbit IgG H&L (1:20000; ab97051; Abcam) for rabbit primary antibodies and Mouse IgG H&L (1:2000; ab6728; Abcam) for mouse primary antibodies. Immunoreactive bands were detected using ECL Prime Western Blotting Detection Reagent (GE Healthcare, Little Chalfont, UK). Protein expression levels were quantified using ImageJ software (National Institutes of Health, Bethesda, MD, USA) and normalized to α-tubulin. The entire Western blot images corresponding to the results presented in Figs. 2, 4, 5 and 6 are provided in the Supplementary Data (Supplementary Figs. S1–S4).

### Statistical analysis

The distribution of continuous variables was assessed using the Shapiro–Wilk test (Supplementary Table S1). All variables except for GPX4 protein expression showed non-normal distribution. Normally distributed data are presented as means ± standard error of the mean (SEM), and non-normally distributed data as medians with ranges. Comparisons among four groups for normally distributed data were performed using one-way analysis of variance (ANOVA) followed by a Tukey’s HSD test, whereas those for non-normally distributed data were performed using the Kruskal–Wallis test followed by a Steel–Dwass post hoc test. We note that, due to the relatively small sample size (*n* = 9–13 per group) and the conservative nature of the Steel–Dwass procedure, the ability to detect significant differences could be lower, potentially increasing the risk of type II errors (false negatives). In addition, because this study primarily focused on the role of renal hL-FABP by comparing hL-FABP Tg and WT mice in RM-induced AKI, when overall group significance was confirmed in the one-way ANOVA or Kruskal–Wallis test, differences between the Tg-RM and WT-RM groups (normalized to each corresponding control) were analyzed using the Student’s t-test for normally distributed data or Mann–Whitney U test for non-normally distributed data. Urinary L-FABP levels (undetectable in WT mice) between the Tg-RM and Tg-Cont groups were compared using the Mann–Whitney U test for non-normally distributed data. For non-normally distributed data, correlations between two parameters were analyzed using Spearman’s rank correlation. Statistical analyses were conducted using JMP software version 17.2.0 (SAS Institute, Cary, NC, USA).

## Results

### Comparison of kidney weight

Both WT and Tg mice in the RM groups exhibited significantly higher normalized kidney weights relative to body weight than their respective control groups on days 1 and 3 after glycerol injection. Although the difference in normalized kidney weights between the Tg-RM and WT-RM groups did not reach significance on either day 1 or 3 in the Steel–Dwass test, the normalized kidney weights in the Tg-RM group were significantly lower than those in the WT-RM group on day 3 according to the Mann–Whitney U test (Table 1).

**Table 1:** Various parameters of day 1 and 3.

Parameter	Day 1				Day 3			
	WT-Cont	WT-RM	Tg-Cont	Tg-RM	WT-Cont	WT-RM	Tg-Cont	Tg-RM
Kidney weight per body weight (g/kg)	12.38 (10.34–13.49)	14.17 (11.90-16.81) ^##^	11.24 (10.70-13.64)	13.98 (10.98–15.49) ^##^	12.24 (10.43–13.06)	18.57 (16.43–20.74) ^##^	12.00 (11.27–14.72)	15.81 (12.02–21.53) ^##^
Relative (fold of Cont)	-	1.17 (0.98–1.39)	-	1.21 (0.95–1.34)	-	1.54 (1.36–1.72)	-	1.29 (0.98–1.75) ^*^
Serum myoglobin (ng/ml)	0.69 (0.52–1.19)	3.14 (1.11–6.02) ^##^	0.78 (0.056–1.95)	2.12 (1.24–9.84) ^##^	0.59 (0.073–1.82)	0.53 (0.25–1.84)	0.95 (0.33–2.15)	0.57 (0.10–1.35)
Relative (fold of Cont)	-	4.18 (1.47–8.01)	-	2.39 (1.40-11.06)	-	0.69 (0.32–2.36)	-	0.54 (0.10–1.28)
Serum cystatin C (µg/ml)	0.61 (0.57–0.81)	1.35 (0.61–1.83) ^##^	0.64 (0.53–0.79)	1.27 (0.91–1.53) ^##^	0.64 (0.56–0.91)	1.58 (1.00-2.74) ^##^	0.65 (0.57–0.77)	0.72 (0.53–1.71)
Relative (fold of Cont)	-	2.05 (0.93–2.79)	-	2.00 (1.43–2.41)	-	2.39 (1.51–4.14)	-	1.10 (0.82–2.63) ^*^
Urinary KIM-1 (ng/day)								
pre-procedure	1.14 (0.29–2.76)	1.24 (0.34–2.61)	0.83 (0.34–3.98)	2.07 (0.73–9.44)	1.18 (0.26–3.24)	1.49 (0.85–5.12)	2.42 (0.94–9.05)	1.89 (0.51–6.88)
post-procedure	1.30 (0.55–1.94)	11.13 (2.51–74.21) ^##^	0.91 (0.055–1.82)	65.74 (0.045–253.20)	1.71 (0.86–2.93)	48.25 (7.65–67.95) ^##^	1.59 (1.14–7.82)	29.09 (9.07–44.72) ^##^
Relative (fold of Cont)	-	8.88 (2.00-59.18)	-	66.55 (0.045–256.30)	-	25.78 (4.09–36.30)	-	11.35 (3.54–17.45) ^*^
Urinary L-FABP (ng/day)								
pre-procedure	-	-	1.02 (0.29–2.47)	1.31 (0.43–5.20)	-	-	1.30 (0.39–3.96)	1.54 (0.52–2.05)
post-procedure	-	-	0.86 (0.052–3.95)	101.33 (5.05-254.46) ^##^	-	-	1.02 (0.40–8.28)	47.86 (6.61-146.56) ^##^

Comparisons among all groups were performed using the Kruskal–Wallis test followed by the Steel–Dwass post hoc test. Differences between Tg-RM and WT-RM groups (normalized to each corresponding control group) were analyzed using the Mann–Whitney U test when Kruskal–Wallis test showed an overall group significance. Urinary L-FABP levels (undetectable in WT mice) between the Tg-RM and Tg-Cont groups were compared using the Mann–Whitney U test for non-normally distributed data. All data are presented as medians with ranges. ^##^ *p* < 0.01 vs. the corresponding group control; * *p* < 0.05 vs. WT-RM.

### Comparison of serum and urinary biochemistry

On day 1, serum myoglobin levels were similarly elevated in the RM groups of both WT and Tg mice compared with their respective control groups. No significant difference in serum myoglobin levels was observed between the Tg-RM and WT-RM groups, as assessed using the Steel–Dwass or Mann–Whitney U test (Table 1). By day 3, no significant differences were observed among all groups. Serum cystatin C levels were significantly higher in the RM groups on day 1, with no notable difference between the Tg-RM and WT-RM groups, as assessed using the Steel–Dwass test or Mann–Whitney U test (Table 1). On day 3, cystatin C levels were significantly higher in the WT-RM group than in the WT-Cont group, but no significant increase was observed in Tg mice. Although the difference in cystatin C levels between the Tg-RM and WT-RM groups was not significant in the Steel–Dwass test, cystatin C levels in the Tg-RM group were significantly lower than those in the WT-RM group according to the Mann–Whitney U test (Table 1).

Regarding urinary biochemistry, urinary KIM-1 levels showed no significant differences between groups before glycerol injection. On day 1 post-injection, urinary KIM-1 levels were significantly higher in the WT-RM group than in the WT-Cont group, with no significant difference between the Tg-RM and Tg-Cont groups. No significant difference in urinary KIM-1 levels was observed between the Tg-RM and WT-RM groups, as assessed using the Steel–Dwass or Mann–Whitney U test (Table 1). By day 3, KIM-1 levels remained higher in the WT-RM group than in the WT-Cont group, but no significant increase was observed in Tg mice. Although the difference in KIM-1 levels between the Tg-RM and WT-RM groups was not significant in the Steel–Dwass test, KIM-1 levels in the Tg-RM group were significantly lower than those in the WT-RM group according to the Mann–Whitney U test (Table 1). Urinary L-FABP levels, measured only in Tg mice, were significantly higher in the Tg-RM group than in the Tg-Cont group on both days 1 and 3 (Table 1).

### Assessment of tubular damage and localization of myoglobin and hL-FABP

PAS-stained specimens revealed significant tubular damage, characterized by tubular dilatation, degeneration, exfoliation of proximal tubular epithelial cells, and cast formation, in the WT-RM and Tg-RM groups on day 1, with no significant differences between them, as assessed using the Steel–Dwass or Mann–Whitney U test (Fig. 1b, d, e). By day 3, tubular damage had worsened in the WT-RM group. Although the difference between the Tg-RM and WT-RM groups was not significant in the Steel–Dwass test, tubular damage in the Tg-RM group was significantly lower than that in the WT-RM group according to the Mann–Whitney U test (Fig. 1c, f, g).

To confirm the presence of myoglobin in renal tissues on day 1—when serum myoglobin levels were similarly elevated in both WT and Tg mice—immunostaining with an anti-myoglobin antibody was conducted (Fig. 2a). Myoglobin-positive areas were detected in the tubules of both RM groups (Fig. 2a). In addition, immunofluorescent staining was performed to determine the localization of hL-FABP (green) and myoglobin (red). In both Tg-Cont and Tg-RM mice, but not WT mice, hL-FABP expression was observed in the proximal tubules (Fig. 2b). In the Tg-RM group, myoglobin was localized within the proximal tubules expressing hL-FABP (Fig. 2b). Furthermore, the fluorescence intensity of hL-FABP was lower in the proximal tubules containing myoglobin than in those without myoglobin (Fig. 2b).

### Analysis of HO-1 expression

On day 1, both mRNA (Fig. 2c, d) and protein (Fig. 2f, h, i) expression levels of HO-1 were significantly higher in the WT-RM and Tg-RM groups than in their respective controls; however, no significant differences in either mRNA or protein expression were observed between those groups using the Steel–Dwass or Mann–Whitney U test. By day 3, HO-1 mRNA expression showed no significant differences among groups (Fig. 2e), whereas HO-1 protein levels remained significantly higher in the WT-RM group than in the WT-Cont group, with no significant difference between the Tg-RM and Tg-Cont groups (Fig. 2g, j). Although the difference in HO-1 protein expression levels between the Tg-RM and WT-RM groups was not significant in the Steel–Dwass test, the expression levels in the Tg-RM group were significantly lower than those in the WT-RM group according to the Mann–Whitney U test (Fig. 2k).

### Evaluation of renal macrophage infiltration and MCP-1 expression

**Fig. 3:**
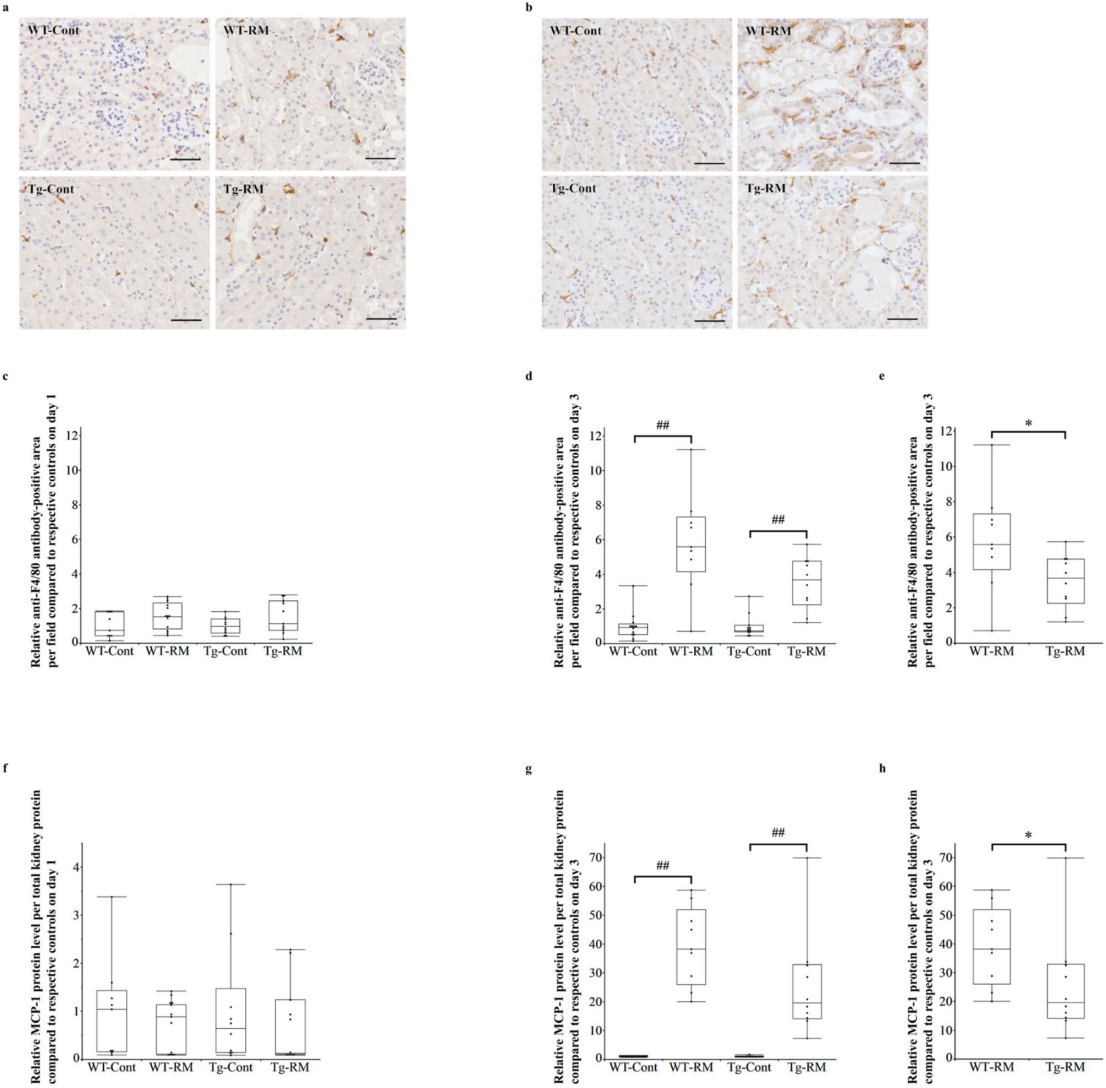
Evaluation of renal macrophage infiltration by immunohistochemical staining for F4/80 and MCP-1 expression by ELISA. (**a**,**b**) Immunohistochemical staining for F4/80 in kidney sections. Representative images of each group on day 1 (**a**) and 3 (**b**). Original magnification: x200. Scale bars = 50 μm. (**c**–**e**) Quantification of F4/80-positive areas on day 1 (**c**) and 3 (**d**,**e**). Data are relative to the respective control groups. (**f**–**h**) ELISA analysis of MCP-1 protein expression in kidney tissues. MCP-1 protein levels were normalized to total kidney protein and relative to the respective control groups on day 1 (**f**) and 3 (**g**,**h**). Comparisons among all groups were performed using the Kruskal–Wallis test followed by the Steel–Dwass post hoc test. Differences between the Tg-RM and WT-RM groups (normalized to each corresponding control group) were analyzed using the Mann–Whitney U test when Kruskal–Wallis test showed an overall group significance. All data are presented as medians with ranges. ^##^*p* < 0.01 vs. the corresponding group control; *p < 0.05 vs. WT-RM.

Immunostaining with an anti-F4/80 antibody revealed the presence of infiltrated macrophages in the renal interstitium on both days 1 and 3 in WT and Tg mice (Fig. 3a, b). On day 1, there were no significant differences in macrophage infiltration between the groups (Fig. 3c). However, by day 3, macrophage infiltration had significantly increased in the WT-RM and Tg-RM groups compared with their respective control groups (Fig. 3d). The Tg-RM group showed significantly lower infiltration than the WT-RM group in the Mann–Whitney U test but not in the Steel–Dwass test (Fig. 3e). In kidney-extracted proteins, MCP-1 expression did not differ significantly among the groups on day 1 (Fig. 3f). However, by day 3, MCP-1 expression was significantly higher in both the WT-RM and Tg-RM groups than in their respective control groups (Fig. 3g). Furthermore, the Tg-RM group exhibited significantly lower MCP-1 expression than the WT-RM group in the Mann–Whitney U test but not in the Steel–Dwass test (Fig. 3h).

### Analysis of NF-κB phosphorylation and PPARα and IκBα expression

**Fig. 4:**
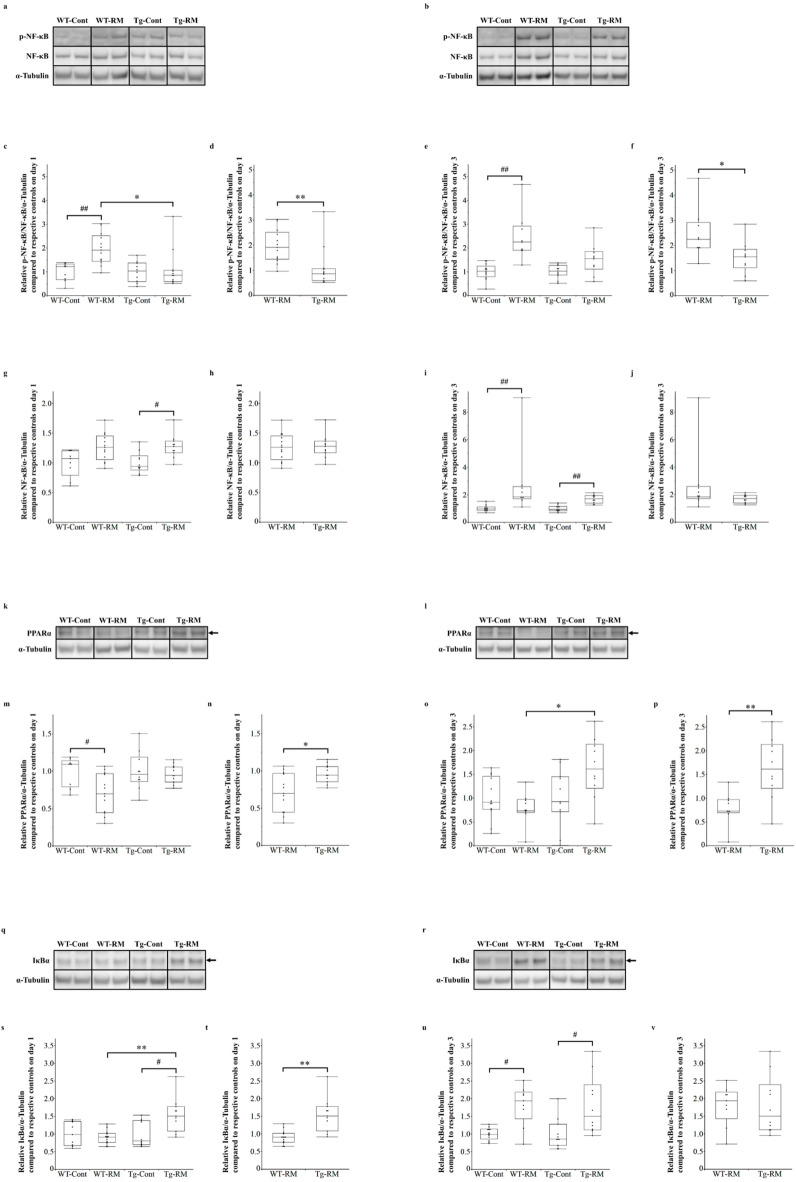
Analysis of NF-κB p65 phosphorylation and PPARα and IκBα expression by Western blotting. (**a**,**b**) Western blot analysis of phosphorylated NF-κB p65 (p-NF-κB) and total NF-κB p65 (NF-κB) in kidney tissues from each group on day 1 (**a**) and 3 (**b**). α-Tubulin was used as a loading control. (**c**–**f**) p-NF-κB/NF-κB quantification normalized to α-Tubulin on day 1 (**c**,**d**) and 3 (**e**,**f**), relative to their respective control groups. (**g**–**j**) Quantification of total NF-κB normalized to α-Tubulin on day 1 (**g**,**h**) and 3 (**i**,**j**), relative to their respective control groups. (**k**,**l**) Western blot analysis of PPARα in kidney tissues from each group on day 1 (**k**) and 3 (**l**). α-Tubulin was used as a loading control. (**m**–**p**) PPARα quantification normalized to α-Tubulin on day 1 (**m**,**n**) and 3 (**o**,**p**), relative to their respective control groups. (**q**,**r**) Western blot analysis of IκBα in kidney tissues from each group on day 1 (**q**) and 3 (**r**). α-Tubulin was used as a loading control. (**s**–**v**) IκBα quantification normalized to α-Tubulin on day 1 (**s**,**t**) and 3 (**u**,**v**), relative to their respective control groups. Arrows indicate the targeted protein. Samples from the same experiment were processed in parallel for SDS polyacrylamide gel electrophoresis (SDS-PAGE) and Western blotting using different gels and membranes, and the image data obtained were cropped. Entire Western blot images are shown in Supplementary Figures S1 and S2. Comparisons among all groups were performed using the Kruskal–Wallis test followed by the Steel–Dwass post hoc test. Differences between Tg-RM and WT-RM groups (normalized to each corresponding control group) were analyzed using the Mann–Whitney U test when Kruskal–Wallis test showed an overall group significance. All data are presented as medians with ranges. ^#^*p* < 0.05 and ^##^*p* < 0.01 vs. the corresponding group control; *p < 0.05 and **p < 0.01 vs. WT-RM.

Western blot analysis detected both phosphorylated NF-κB and total NF-κB expression on days 1 (Fig. 4a) and 3 (Fig. 4b). On day 1, NF-κB phosphorylation was significantly higher in the WT-RM group than in the WT-Cont group, whereas the Tg-RM group showed significantly reduced phosphorylation compared with the WT-RM group in the Steel–Dwass and Mann–Whitney U tests (Fig. 4c, d). By day 3, phosphorylation levels remained elevated in the WT-RM group compared with the WT-Cont group, with no significant difference between the Tg-RM and Tg-Cont groups (Fig. 4e). Although the difference in phosphorylation levels between the Tg-RM and WT-RM groups was not significant in the Steel–Dwass test, the phosphorylation levels in the Tg-RM group were significantly lower than those in the WT-RM group according to the Mann–Whitney U test (Fig. 4e, f). Regarding total NF-κB expression, the Tg-RM group showed significantly higher levels than the Tg-Cont group on day 1, with no significant difference between the WT-RM and WT-Cont groups (Fig. 4g). By day 3, total NF-κB expression was significantly elevated in both the WT-RM and Tg-RM groups compared with their respective controls (Fig. 4i). However, both the Steel–Dwass and Mann–Whitney U tests showed no significant differences between the WT-RM and Tg-RM groups on day 1 or 3 (Fig. 4g–j).

Western blotting was used to evaluate PPARα expression levels (Fig. 4k–p). On day 1 (Fig. 4k, m, n), PPARα expression was significantly lower in the WT-RM group than in the WT-Cont group, whereas no significant difference was observed between the Tg-RM and Tg-Cont groups (Fig. 4m). Although no significant difference was found between the WT-RM and Tg-RM groups in the Steel–Dwass test (Fig. 4m), the PPARα expression in the Tg-RM group was significantly higher than that in the WT-RM group according to the Mann–Whitney U test (Fig. 4n). By day 3 (Fig. 4l, o, p), the Steel–Dwass and Mann–Whitney U tests showed significantly higher PPARα expression in the Tg-RM group than in the WT-RM group (Fig. 4o, p).

To investigate the upstream regulation of the NF-κB pathway, IκBα expression was assessed via Western blotting on days 1 (Fig. 4q) and 3 (Fig. 4r). On day 1, IκBα expression was significantly higher in the Tg-RM group than in the Tg-Cont group, whereas the Tg-RM group showed significantly higher expression than the WT-RM group in both the Steel–Dwass and Mann–Whitney U tests (Fig. 4s, t). By day 3, IκBα expression was significantly increased in the WT-RM and Tg-RM groups compared with their respective controls (Fig. 4u). However, neither the Steel–Dwass nor Mann–Whitney U test detected significant differences between the WT-RM and Tg-RM groups (Fig. 4u, v).

### Analysis of lipid peroxidation

**Fig. 5:**
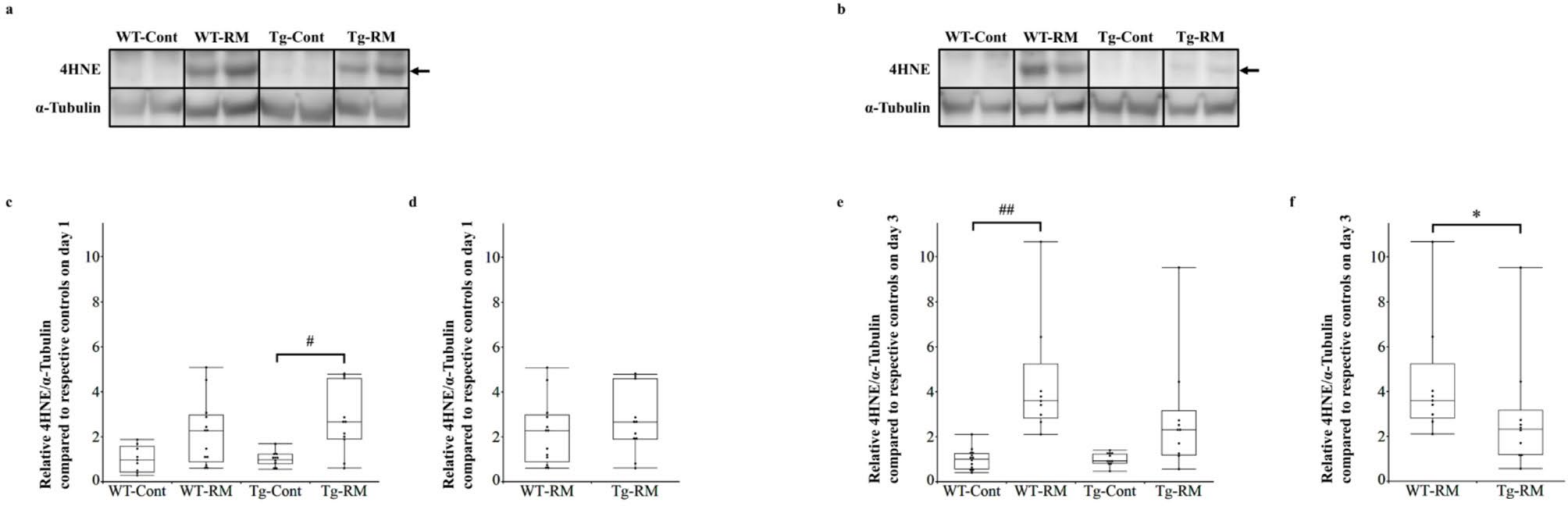
4HNE expression analysis by Western blotting. (**a**,**b**) Western blot analysis of 4HNE in kidney tissues from each group on day 1 (**a**) and 3 (**b**). α-Tubulin was used as a loading control. (**c**–**f**) 4HNE quantification normalized to α-Tubulin on day 1 (**c**,**d**) and 3 (**e**,**f**), relative to their respective control groups. Arrows indicate the targeted protein. Samples from the same experiment were processed in parallel for SDS-PAGE and Western blotting using different gels and membranes, and the image data obtained were cropped. Entire Western blot images are shown in Supplementary Figure S3. Comparisons among all groups were performed using the Kruskal–Wallis test followed by the Steel–Dwass post hoc test. Differences between Tg-RM and WT-RM groups (normalized to each corresponding control group) were analyzed using the Mann–Whitney U test when Kruskal–Wallis test showed an overall group significance. All data are presented as medians with ranges. ^#^*p* < 0.05 and ^##^*p* < 0.01 vs. the corresponding group control; *p < 0.05 vs. WT-RM.

Western blotting with an anti-4HNE antibody was conducted to evaluate lipid peroxidation in renal tissues (Fig. 5a, b). On day 1, 4HNE expression was significantly higher in the Tg-RM group than in the Tg-Cont group, whereas no significant difference was observed between the WT-RM and WT-Cont groups (Fig. 5c). By day 3, 4HNE expression was significantly higher in the WT-RM group than in the WT-Cont group (Fig. 5e). The Steel–Dwass test did not detect significant differences between the WT-RM and Tg-RM groups on either day 1 (Fig. 5c) or 3 (Fig. 5e). However, the Mann–Whitney U test showed significantly lower 4HNE expression in the Tg-RM group than in the WT-RM group on day 3, but not on day 1 (Fig. 5d, f).

### Analysis of ferroptosis-related molecules

**Fig. 6:**
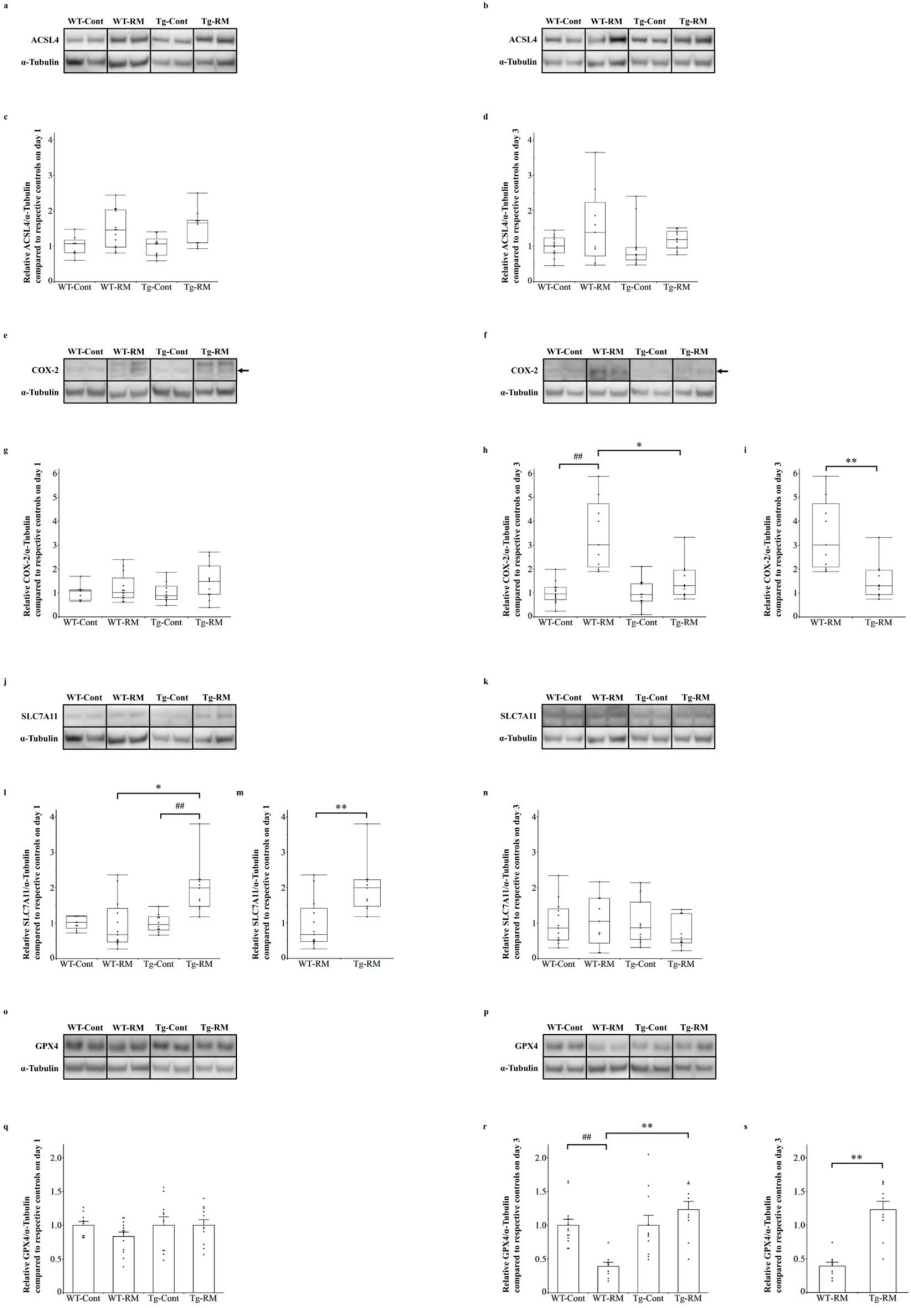
Analysis of ferroptosis-related molecules by Western blotting. (**a**–**d**) Representative Western blot images and quantification of ACSL4 protein levels normalized to α-Tubulin on day 1 (**a**,**c**) and 3 (**b**,**d**), relative to their respective control groups. (**e**–**i**) Representative Western blot images and quantification of COX-2 protein levels normalized to α-Tubulin on day 1 (**e**,**g**) and 3 (**f**,**h**,**i**), relative to their respective control groups. Arrows indicate the targeted protein. (**j**–**n**) Representative Western blot images and quantification of SLC7A11 protein levels normalized to α-Tubulin on day 1 (**j**,**l**,**m**) and 3 (**k**,**n**), relative to their respective control groups. (**o**–**s**) Representative Western blot images and quantification of GPX4 protein levels normalized to α-Tubulin on day 1 (**o**,**q**) and 3 (**p**,**r**,**s**), relative to their respective control groups. Samples from the same experiment were processed in parallel for SDS-PAGE and Western blotting using different gels and membranes, and the image data obtained were cropped. Entire Western blot images are shown in Supplementary Figure S4. Comparisons among all groups were performed using one-way ANOVA followed by Tukey’s HSD test for GPX4 data, or the Kruskal–Wallis test followed by the Steel–Dwass post hoc test for ACSL4, COX-2, and SLC7A11 data. Differences between Tg-RM and WT-RM groups (relative to their controls) were assessed with Student’s t-test after significant one-way ANOVA for GPX4, or Mann–Whitney U test after significant Kruskal–Wallis test for ACSL4, COX-2, and SLC7A11. ACSL4, COX-2, and SLC7A11 data are shown as medians with ranges, and GPX4 data as the mean ± SEM. ^##^*p* < 0.01 vs. the corresponding group control; *p < 0.05 and **p < 0.01 vs. WT-RM.

Western blotting was used to evaluate the expression levels of ACSL4 (Fig. 6a–d), COX-2 (Fig. 6e–i), SLC7A11 (Fig. 6j–n), and GPX4 (Fig. 6o–s), which are associated with ferroptosis^13,14^. No significant differences in ACSL4 protein expression were observed among the groups on days 1 (Fig. 6a, c) and 3 (Fig. 6b, d).

For COX-2 protein expression, no significant differences were observed among the groups on day 1 (Fig. 6e, g). However, on day 3, COX-2 expression levels were significantly higher in the WT-RM group than in the WT-Cont group (Fig. 6f, h). The Tg-RM group showed significantly lower expression than the WT-RM group, as confirmed via the Steel–Dwass and Mann–Whitney U tests (Fig. 6h, i).

SLC7A11 protein expression levels were significantly higher in the Tg-RM group than in the Tg-Cont group on day 1 (Fig. 6j, l). In addition, the Tg-RM group exhibited significantly higher expression than the WT-RM group, as confirmed via the Steel–Dwass and Mann–Whitney U tests (Fig. 6l, m). However, no significant differences were observed among the groups on day 3 (Fig. 6k, n).

Regarding GPX4 protein expression, no significant differences were observed among the groups on day 1 (Fig. 6o, q). On day 3, GPX4 expression was significantly lower in the WT-RM group than in the WT-Cont group, but this reduction was not observed in the Tg-RM group relative to the Tg-Cont group (Fig. 6p, r). In addition, a significant difference in GPX4 expression was observed between the WT-RM and Tg-RM groups, as confirmed via the Steel–Dwass and Mann–Whitney U tests (Fig. 6r, s).

### Relationship between urinary hL-FABP and degree of tubular damage, macrophage infiltration, and lipid peroxidation

**Fig. 7:**
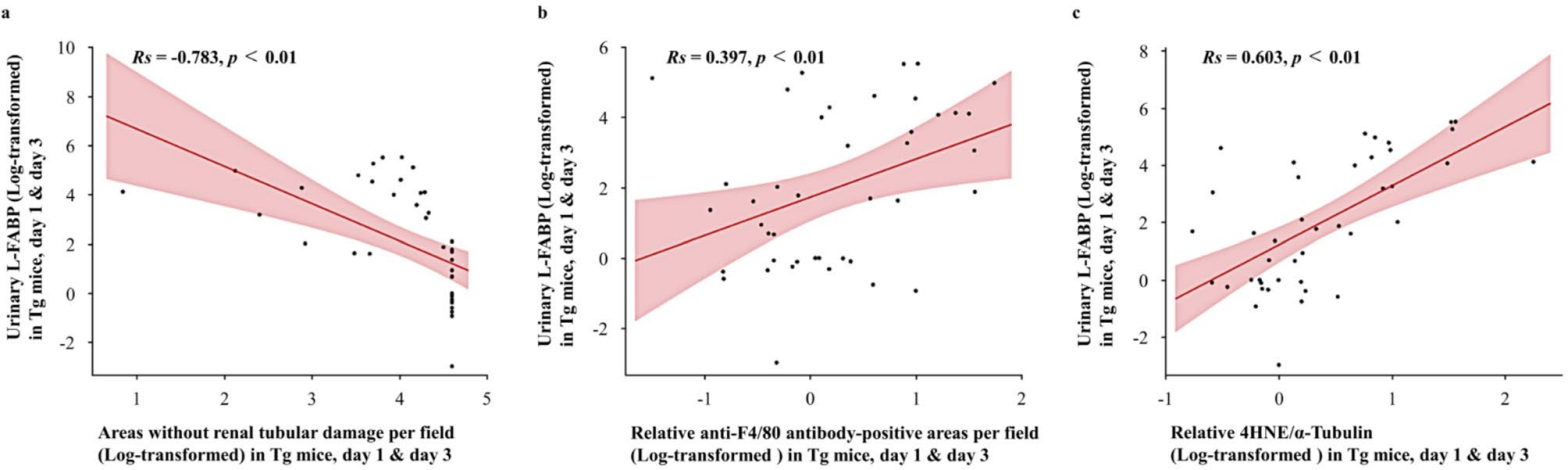
Correlation between urinary L-FABP levels on days 1 and 3 in Tg mice. (**a**–**c**) Correlation of urinary L-FABP levels with the percentage of areas without renal tubular damage per field (**a**), F4/80-positive areas (**b**), and quantification of 4HNE protein levels normalized to α-Tubulin (**c**). All values are log-transformed. Correlations were analyzed using Spearman’s rank correlation.

Urinary L-FABP levels demonstrated a significant negative correlation with the percentage of areas without renal tubular damage (Rs = − 0.783, *p* < 0.01, Fig. 7a). In addition, there was a significant positive correlation between urinary L-FABP levels and both the degree of macrophage infiltration (Rs = 0.397, *p* < 0.01, Fig. 7b) and 4-HNE expression (Rs = 0.603, *p* < 0.01, Fig. 7c).

## Discussion

This study demonstrated that after intramuscular glycerol injection into the quadriceps, Tg mice exhibited significant suppression of kidney enlargement, renal dysfunction, urinary KIM-1 elevation, morphological tubular damage, macrophage infiltration, increased MCP-1 expression, and NF-κB activation, all of which were observed in WT mice. On the other hand, serum myoglobin levels and HO-1 mRNA and protein expression levels on day 1 remained comparable between the Tg-RM and WT-RM groups. Under conditions of uncontrolled oxidative stress caused by myoglobin reabsorption in the tubules, the increased protein expression of both the oxidative stress marker HO-1 and the lipid peroxidation marker 4HNE in WT-RM mice on day 3 was not observed in Tg-RM mice. This suppression led to a reduction of ferroptosis, as evidenced by the inhibition of COX-2 upregulation and preservation of GPX4 expression. The colocalization of hL-FABP expression and myoglobin in kidney tissue suggests that renal hL-FABP may confer renoprotective effects by modulating ferroptosis through its antioxidant activity against oxidative stress induced by myoglobin in RM-induced AKI.

Among the various molecular mechanisms involved in RM-induced AKI, oxidative stress caused by the generation of reactive oxygen species leads to ferroptosis, which is dependent on lipid peroxidation resulting from excessive iron accumulation and plays a key role in worsening AKI^1,15^. GPX4 is a major inhibitor of ferroptosis as it prevents the build-up of toxic lipid peroxidation products by converting lipid hydroperoxides into non-toxic lipid alcohols using glutathione (GSH) as a substrate^16,17^. Notably, Tg-RM mice significantly inhibited the downregulation of GPX4 expression observed in WT-RM mice on day 3. Regarding other ferroptosis-related molecules, Tg-RM mice on days 1 and 3 did not show any alteration in the upregulated expression of ACSL4, which enhances susceptibility to oxidative stress in polyunsaturated fatty acids and contributes to the progression of ferroptosis. However, they exhibited a marked increase in the expression of SLC7A11, a transporter located in the cell membrane that facilitates intracellular GSH synthesis for GPX4 function^16,18^. In addition, Tg-RM mice showed a downregulation in COX-2 expression, which is considered a marker of ferroptosis despite not directly oxidizing phospholipids^13^. As ferroptosis depends on a balance of proferroptotic and antiferroptotic factors, this molecular profile suggests that Tg-RM mice have lower membrane damage and ferroptosis susceptibility than WT-RM mice, even with similar ACSL4 levels. Based on these findings, renal hL-FABP appears to be involved in reducing ferroptosis in RM-induced AKI.

Previous experimental studies using hL-FABP Tg mice from this study reported that renal hL-FABP is expressed in proximal tubules and plays a crucial role in alleviating tubular damage by reducing oxidative stress in various acute kidney diseases^8,9,11^. This role was also supported in RM-induced AKI. It has been suggested that hL-FABP may act as a scavenger of lipid peroxidation products due to its high affinity for fatty acids with long alkyl chains, carbon double bonds or fatty acid peroxides, thereby mitigating oxidative damage^19^. To the best of our knowledge, the involvement of renal L-FABP in ferroptosis and its role in RM-induced AKI have not yet been investigated; thus, this study provides the first evidence that renal hL-FABP, through its antioxidative function, mitigates RM-induced AKI by inhibiting ferroptosis.

NF-κB activation is triggered by oxidative stress and plays a key role in promoting inflammation, including the upregulation of MCP-1 and COX-2 expression and macrophage infiltration into the interstitium^20^. In this study, NF-κB inactivation coupled with upregulated IκBα expression on days 1 and 3 in Tg-RM mice may have contributed to reduced renal inflammation and prevented macrophage infiltration on day 3. However, while 4HNE levels in Tg-RM mice were significantly higher than those in Tg-Cont mice and not significantly different from those in WT-RM mice on day 1, NF-κB activation was significantly suppressed in Tg-RM mice on the same day. Since L-FABP is suggested not only to easily bind to lipid peroxides for detoxification but also to act as a scavenger of free radicals^19,21^, the inactivation of NF-κB in Tg-RM mice on day 1 may have resulted from free radical reduction by hL-FABP, potentially leading to the decreased 4HNE levels observed on day 3. In addition, L-FABP has been reported to be involved in the activation of PPARα, which in turn suppresses NF-κB activation^22,23^. The present study showed higher PPARα expression in Tg-RM mice than in WT-RM mice, but no significant upregulation in Tg-RM mice compared with Tg-Cont mice. Based on these findings, we speculate that renal hL-FABP may have inhibited NF-κB activation on day 1 by maintaining PPARα expression. Further research is needed to clarify how renal hL-FABP contributes to early NF-κB inactivation in RM-induced AKI.

Accumulating evidence supports the clinical utility of urinary hL-FABP for the early diagnosis of AKI and prognosis prediction^24–27^. In preclinical studies using hL-FABP Tg mice, urinary hL-FABP has been shown to accurately reflect the extent of tubular damage in various AKI models, including those induced by ischemia–reperfusion^6,8–11^, medication^9^, folic acid^28^, and histones accompanied with severe infection^29^. However, its role in RM-induced AKI had not been previously evaluated. This study newly demonstrated the relationship between urinary hL-FABP and the degree of tubular damage, macrophage infiltration, and lipid peroxidation in RM-induced AKI (Fig. 7). In ischemia–reperfusion AKI, hL-FABP translocates from the cytoplasm to the tubular lumen of proximal tubular cells, leading to increased urinary hL-FABP levels^6,8–11^. Similarly, in RM-induced AKI, hL-FABP expression was lower in myoglobin-positive tubules than in myoglobin-negative tubules, and urinary hL-FABP levels increased in Tg-RM mice, mirroring findings from the ischemia–reperfusion model. These results suggest that in RM-induced AKI, hL-FABP may be excreted into the urine in proportion to AKI severity, making urinary hL-FABP a potential biomarker for assessing RM-induced AKI severity.

In this study, higher levels of phosphorylated NF-κB together with upregulated IκBα expression were observed in WT-RM mice on day 3. The upregulation of IκBα expression is considered to represent negative feedback to the increased phosphorylated NF-κB levels in the WT-RM group at this time point^30,31^.

There is a critical limitation in this study. While no significant difference was detected between the Tg-RM and WT-RM groups in four-group comparisons using the Steel–Dwass test, significant differences emerged in two-group analyses (Mann–Whitney U test). This discrepancy suggests a modest renoprotective effect of hL-FABP against RM-induced AKI, thus requiring cautious interpretation.

In conclusion, this study demonstrated that renal hL-FABP exerts renoprotective effects in RM-induced AKI, at least in part, by alleviating oxidative stress and suppressing ferroptosis. These findings suggest that renal hL-FABP may be a potential therapeutic target for preventing the progression of RM-induced AKI.

## Supplementary Information

Below is the link to the electronic supplementary material.

Supplementary Material 1

## Data Availability

The data used to support the findings of this study are included within the article.
