# Supplementary material for: Renoprotective effects of human L-type fatty acid-binding protein (hL-FABP) in rhabdomyolysis-induced acute kidney injury
Source: Sci Rep. 2025 Dec 26;16:3940. doi: 10.1038/s41598-025-34045-9 (PMC12855215; doi:10.1038/s41598-025-34045-9)
Supplement: Supplementary file 1 — Supplementary Material 1 [file 41598_2025_34045_MOESM1_ESM.pdf]

Renoprotective effects of human L-type fatty acid-binding protein (hL-FABP) in rhabdomyolysis-induced acute kidney injury

Kazuho Inoue[1], Seiko Hoshino[1], Keiichi Ohata[2], Takeshi Sugaya[2], Kenjiro Kimura[3], Yugo Shibagaki[2], Atsuko Kamijo-Ikemori[1, 2, 4, *]

1. Department of Anatomy, St. Marianna University School of Medicine, Kanagawa, Japan.

2. Division of Nephrology and Hypertension, Department of Internal Medicine, St. Marianna University School of Medicine, Kanagawa, Japan.

3. JCHO Tokyo Takanawa Hospital, Tokyo, Japan.

4. Institute for Animal Experimentation, St. Marianna University Graduate School of Medicine, Kanagawa, Japan.

*Corresponding author

Corresponding author: Atsuko Kamijo-Ikemori, M.D., Ph.D.

Department of Anatomy, and Division of Nephrology and Hypertension, Department of Internal Medicine, St. Marianna University School of Medicine

2-16-1 Sugao, Miyamae-Ku, Kawasaki 216-8511, Japan

Tel: +81-44-977-8111 ext. 3630, Fax: +81-44-976-7083, E-mail: a2kamijo@marianna-u.ac.jp

Supplementary data

Supplementary Table S1. Shapiro–Wilk test for evaluating whether each variable follows a normal distribution.

Variable	Shapiro–Wilk test statistic (W)	p-value	Normal / Non-normal
kidney weight / Body weight	0.8741417	<0.0001	Non-normal
Serum myoglobin	0.6998401	<0.0001	Non-normal
Serum cystatin C	0.8117997	<0.0001	Non-normal
Urinary KIM-1 pre-procedure	0.1531072	<0.0001	Non-normal
Urinary KIM-1 post-procedure	0.5797271	<0.0001	Non-normal
Urinary L-FABP pre-procedure	0.8373071	<0.0001	Non-normal
Urinary L-FABP post-procedure	0.6789106	<0.0001	Non-normal
Percentage of areas without renal tubular damage per field	0.7736809	<0.0001	Non-normal
Relative mRNA expression of HO-1/18S compared to respective controls	0.7521328	<0.0001	Non-normal
Relative HO-1/α-Tubulin compared to respective controls	0.8875911	<0.0001	Non-normal
Relative anti-F4/80 antibody-positive area per field compared to respective controls	0.7586983	<0.0001	Non-normal
Relative MCP-1 protein level per total kidney protein compared to respective controls	0.5606884	<0.0001	Non-normal
Relative p-NF-κB/NF-κB/α-Tubulin compared to respective controls	0.8874216	<0.0001	Non-normal
Relative NF-κB/α-Tubulin compared to respective controls	0.4284216	<0.0001	Non-normal
Relative PPARα/α-Tubulin compared to respective controls	0.9524025	0.0031	Non-normal
Relative IκB/α-Tubulin compared to respective controls	0.8739897	<0.0001	Non-normal
Relative 4HNE/α-Tubulin compared to respective controls	0.734651	<0.0001	Non-normal
Relative ACSL4/α-Tubulin compared to respective controls	0.8921152	<0.0001	Non-normal
Relative COX-2/α-Tubulin compared to respective controls	0.77683	<0.0001	Non-normal
Relative SLC7A11/α-Tubulin compared to respective controls	0.9180549	<0.0001	Non-normal
Relative GPX4/α-Tubulin compared to respective controls	0.9841102	0.3738	Normal

Shapiro–Wilk test results for all measured variables. The column “Shapiro–Wilk test statistic (W)” shows the test statistic, and the “p-value” column indicates statistical significance. Variables with $p \geq 0.05$ were classified as normally distributed, while those with $p < 0.05$ were classified as non-normal.

Supplementary Figures

Entire images of Western Blotting

Figure S1

A. HO-1

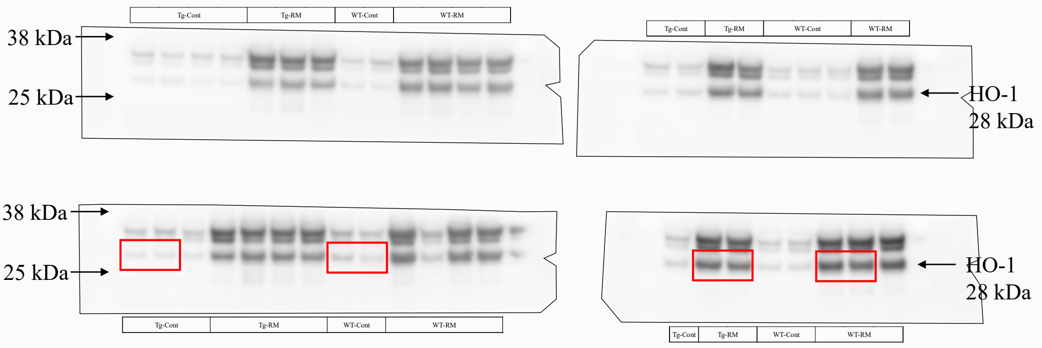

B. PPARα

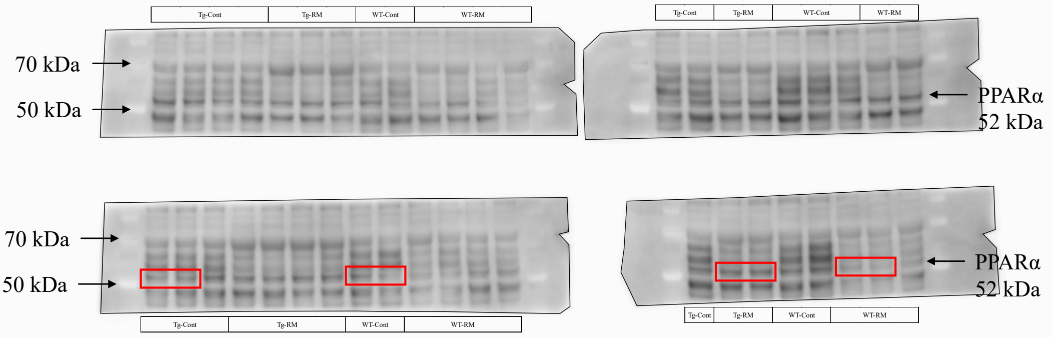

C. α-Tubulin for A and B from the same membrane

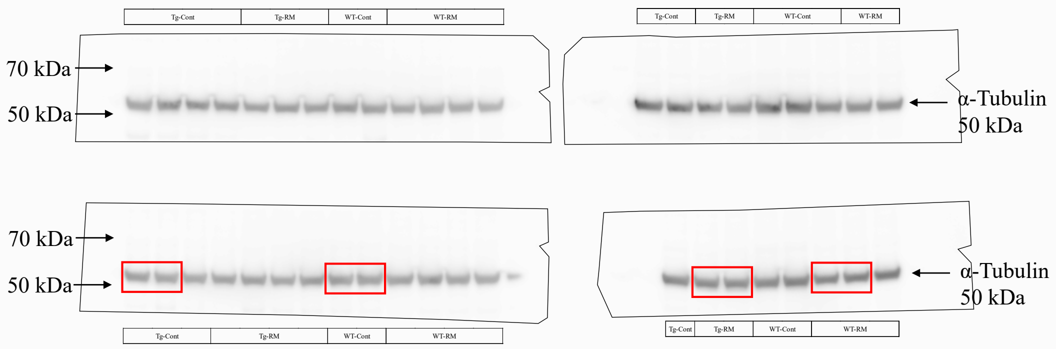

D. HO-1

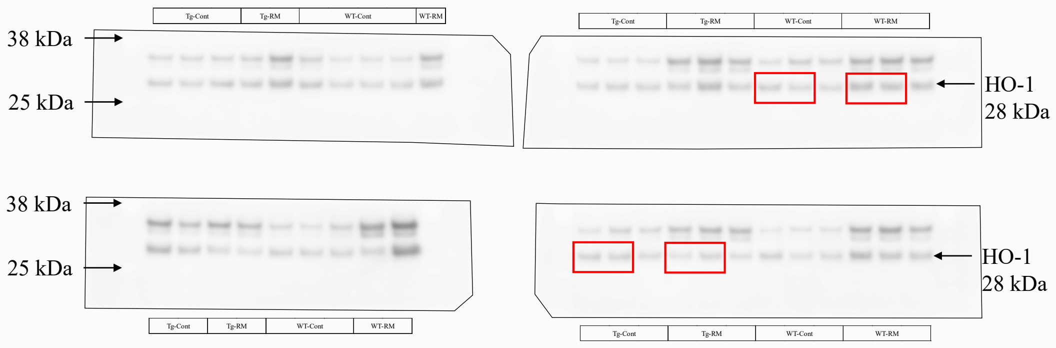

E. PPARα

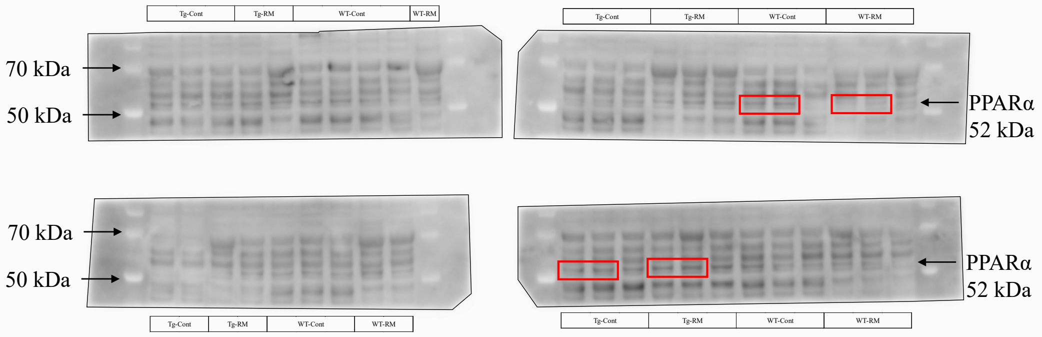

F. α-Tubulin for D and E from the same membrane

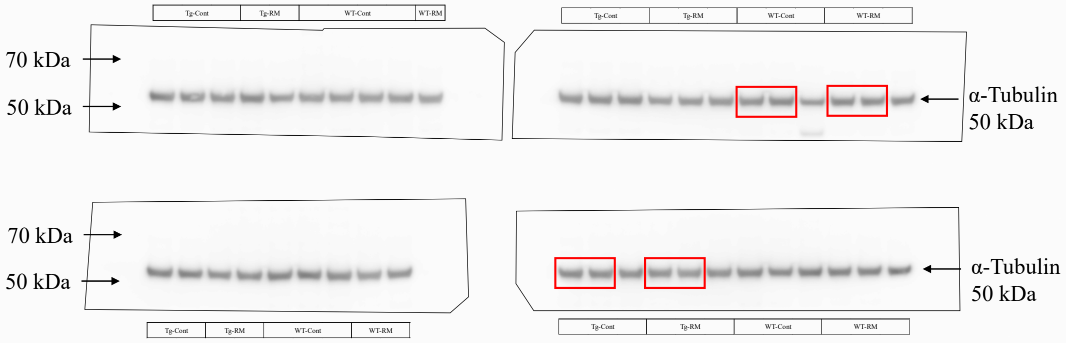

Western blotting of HO-1, PPARα and α-Tubulin on day 1 (A-C) and day 3 (D-F). Black lines represent the edge of each cut membrane. Red boxes show the regions of the original blots used in main figures.

Figure S2

A. p-NF-κB

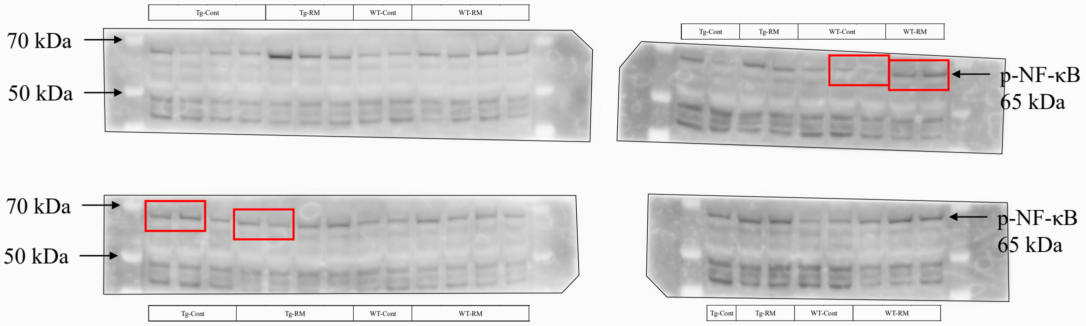

B. NF-κB

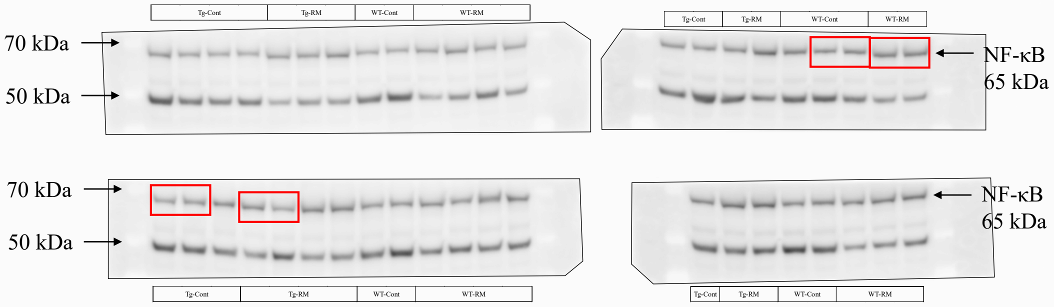

C. α-Tubulin for A and B from the same membrane

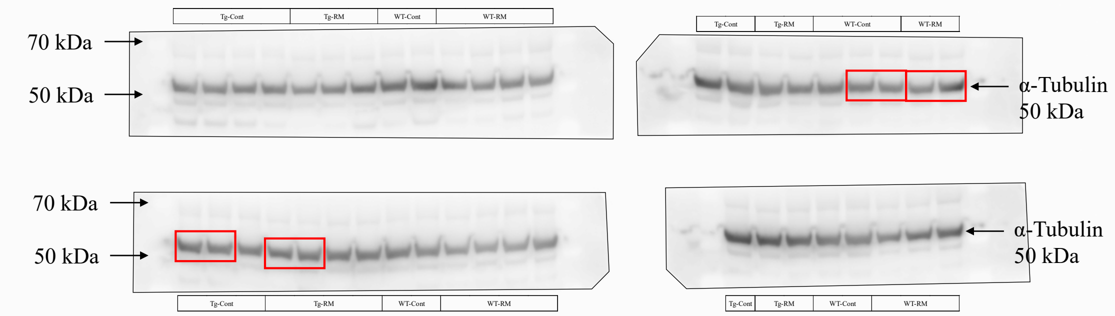

D. IκBα

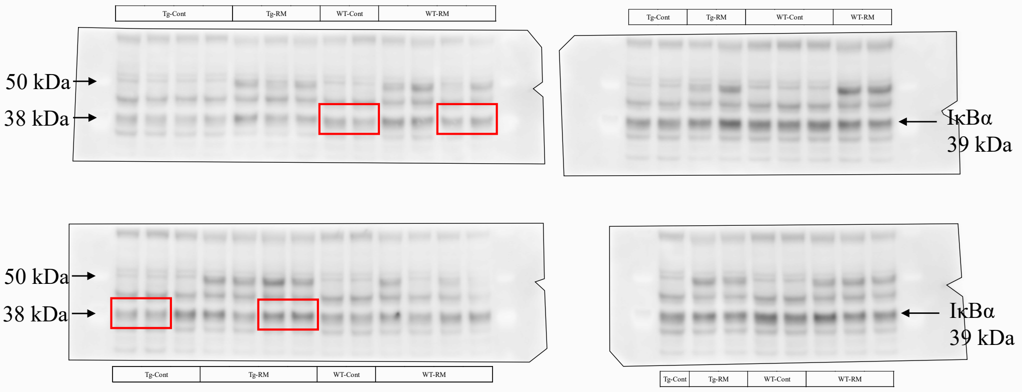

E. α-Tubulin for D from the same membrane

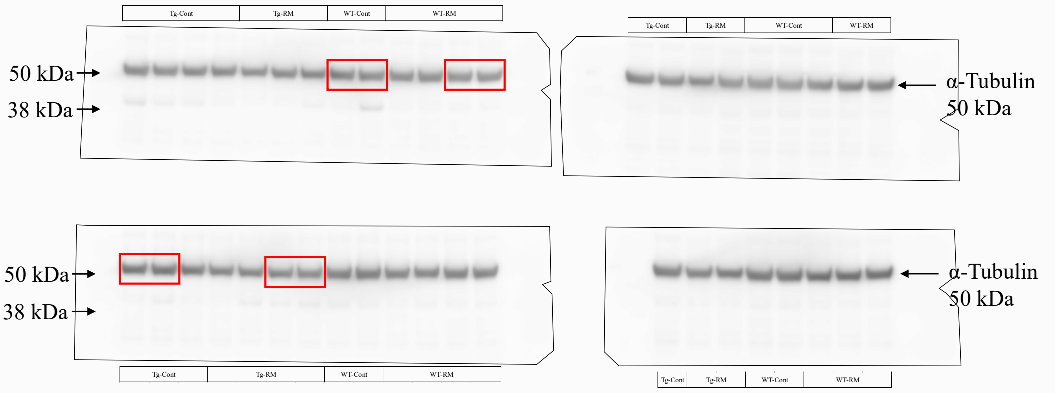

F. p-NF-κB

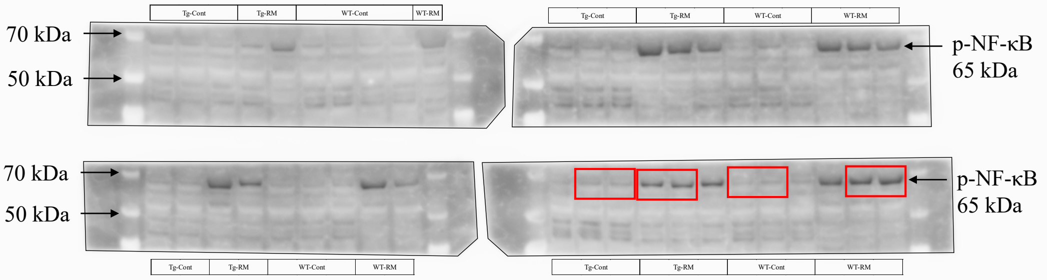

G. NF-κB

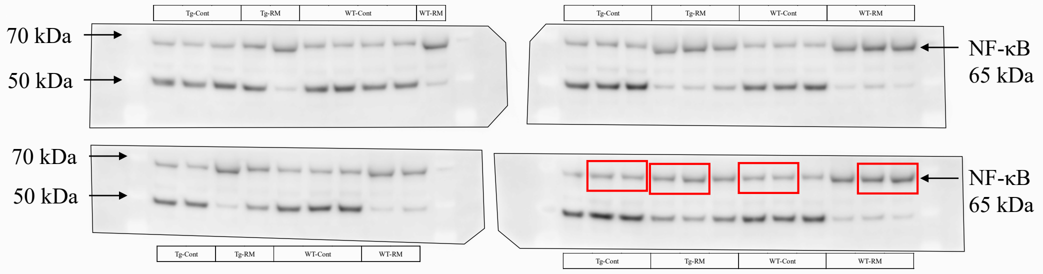

H. α-Tubulin for F and G from the same membrane

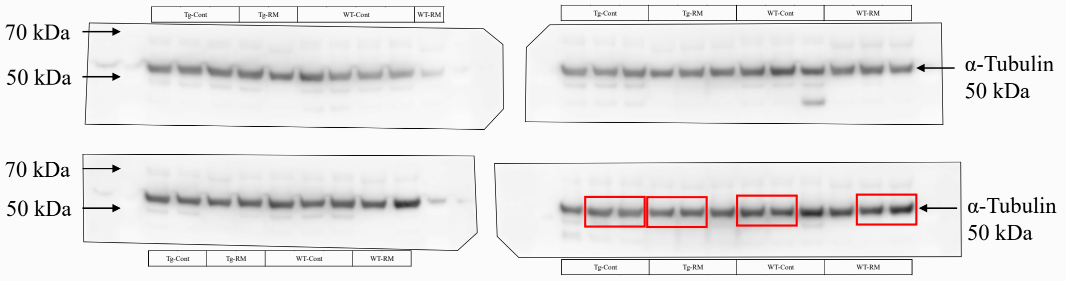

I. IκBα

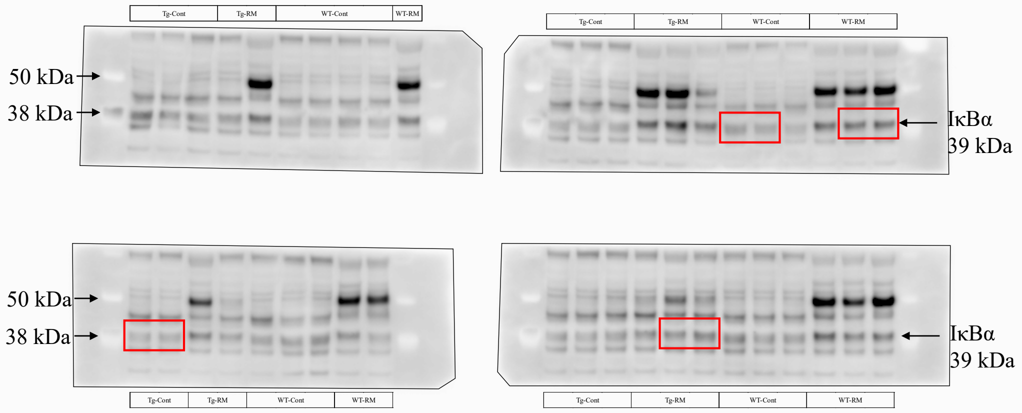

J. α-Tubulin for I from the same membrane

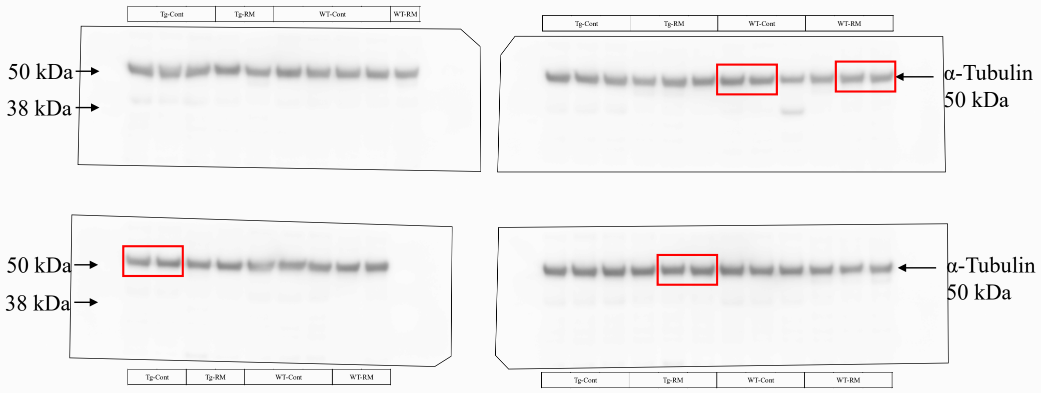

Western blotting of p-NF-κB, NF-κB, IκBα and α-Tubulin on day 1 (A-E) and day 3 (F-J). Black lines represent the edge of each cut membrane. Red boxes show the regions of the original blots used in main figures.

Figure S3

A. 4HNE

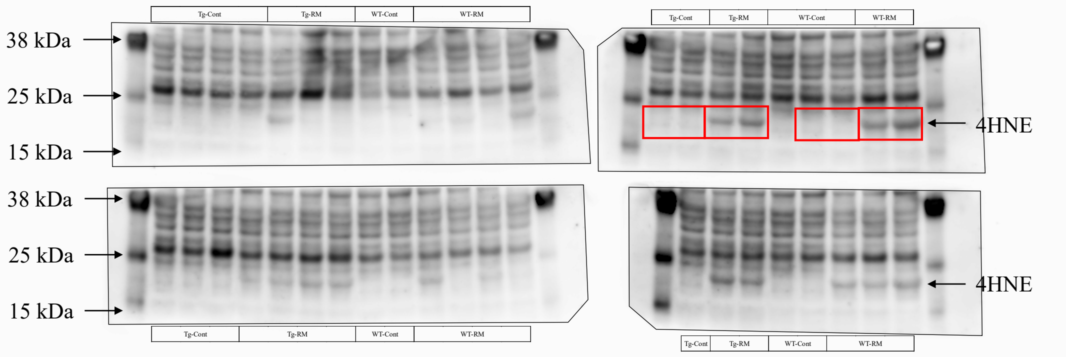

B. α-Tubulin for A from the same membrane

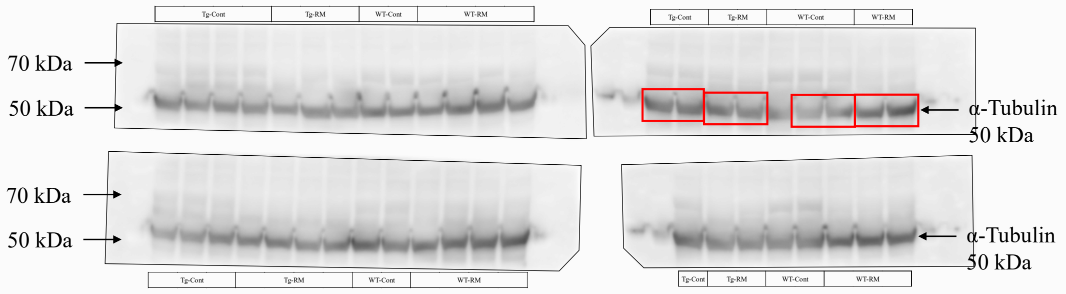

C. 4HNE

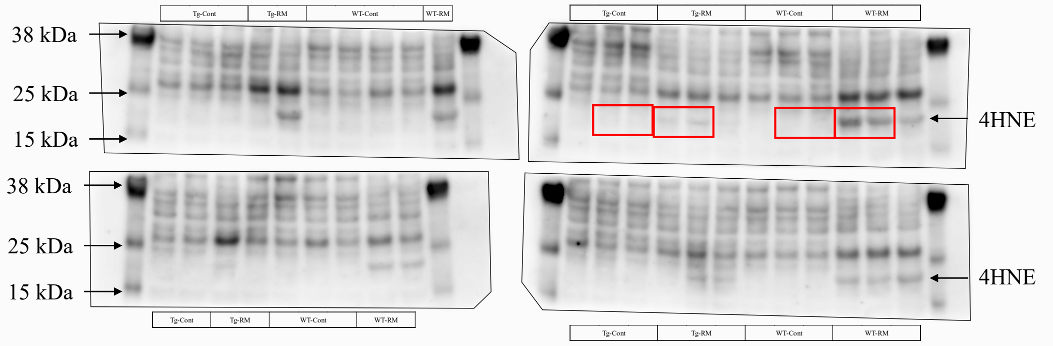

D. α-Tubulin for C from the same membrane

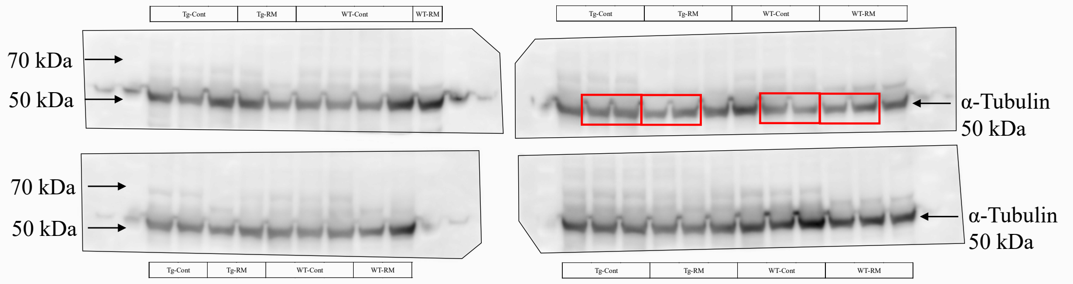

Western blotting of 4HNE and α-Tubulin on day 1 (A and B) and day 3 (C and D). Black lines represent the edge of each cut membrane. Red boxes show the regions of the original blots used in main figures.

Figure S4

A. ACSL4

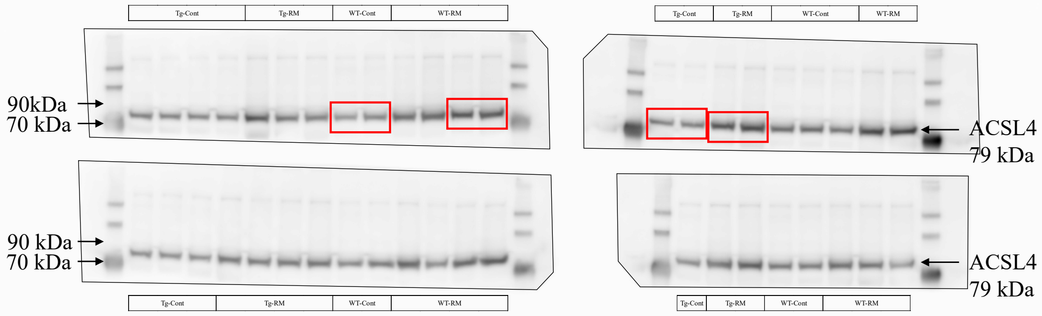

B. SLC7A11

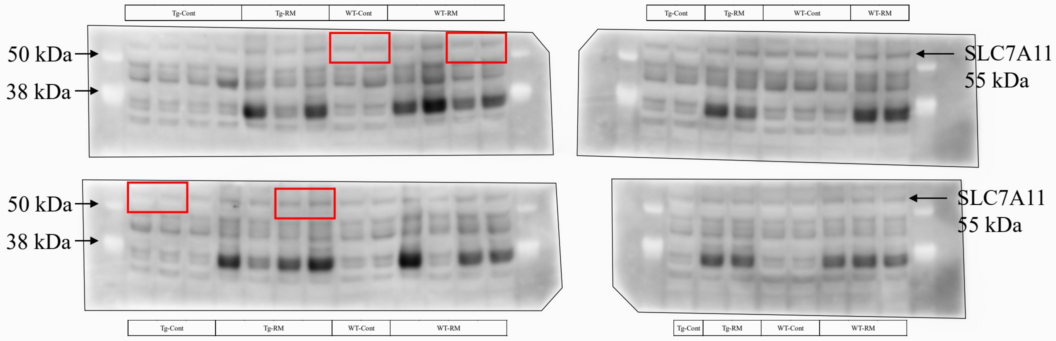

C. α-Tubulin for A and B from the same membrane

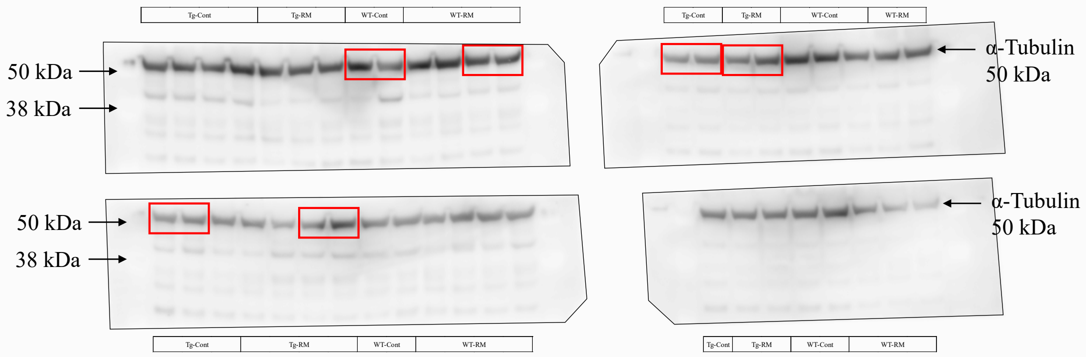

D. COX-2

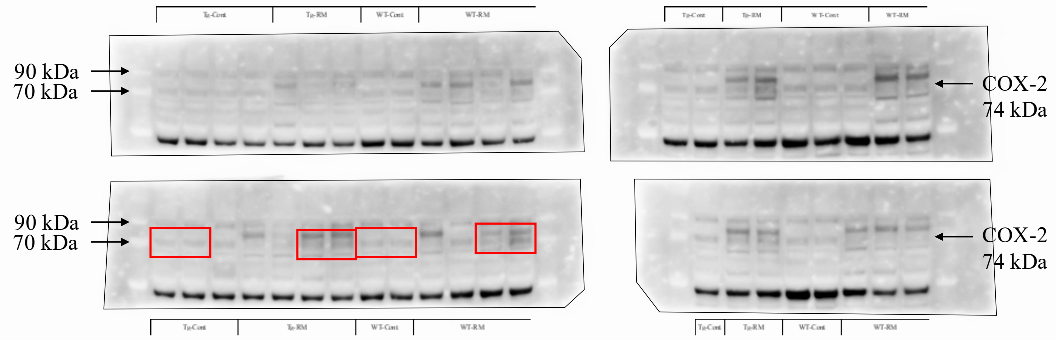

E. α-Tubulin for D from the same membrane

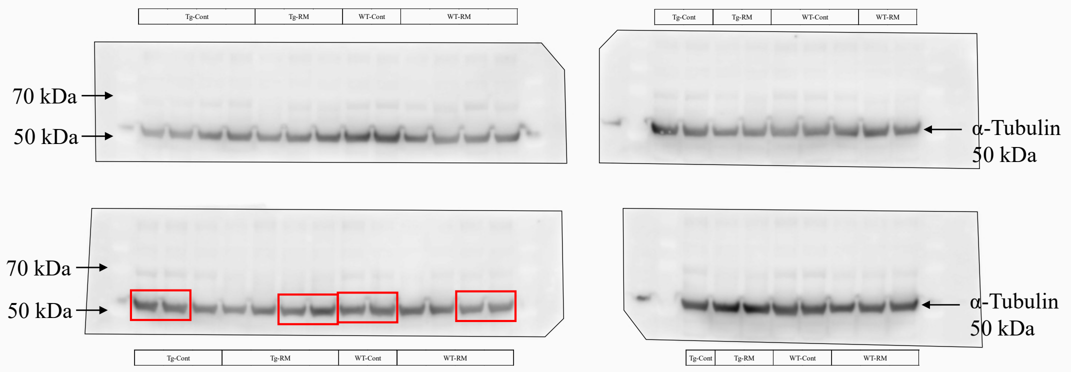

F. GPX4

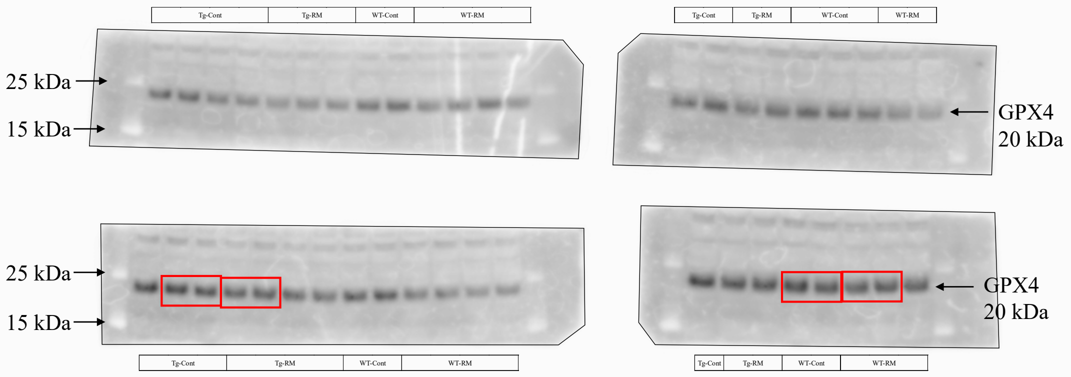

G. α-Tubulin for F from the same membrane

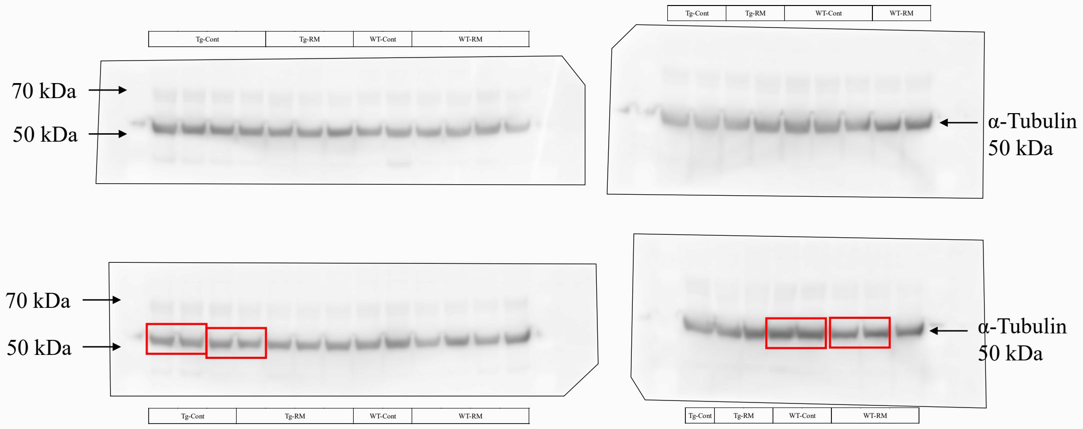

H. ACSL4

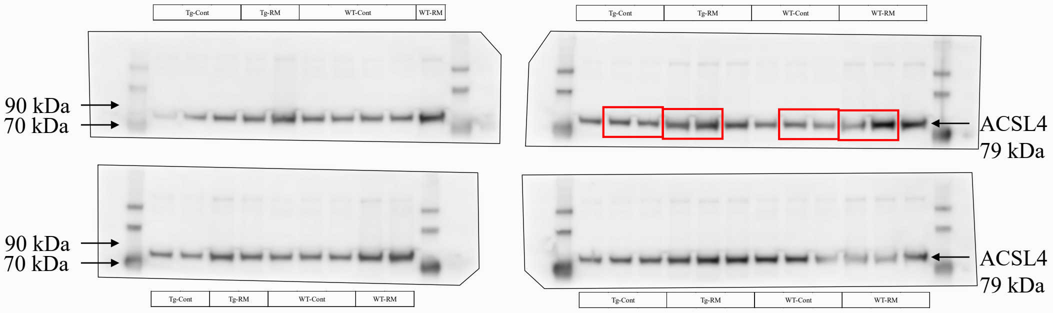

I. SLC7A11

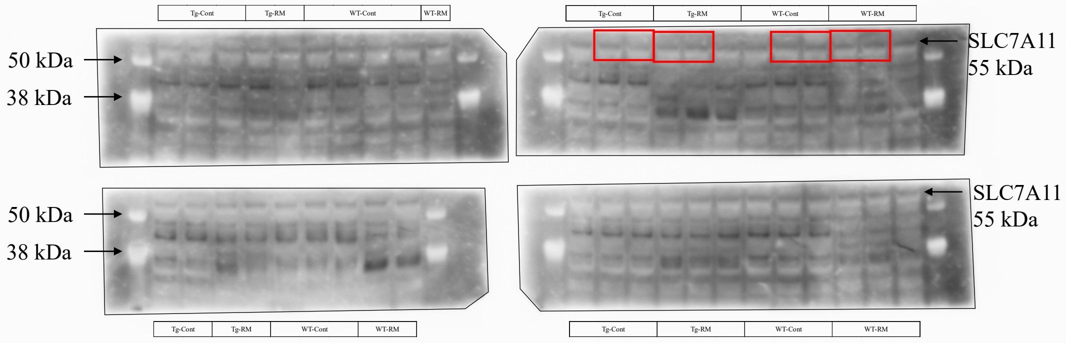

J. α-Tubulin for H and I from the same membrane

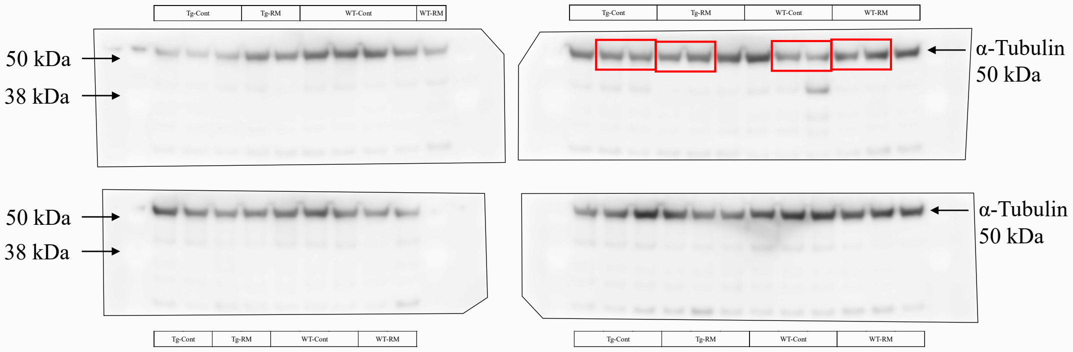

K. COX-2

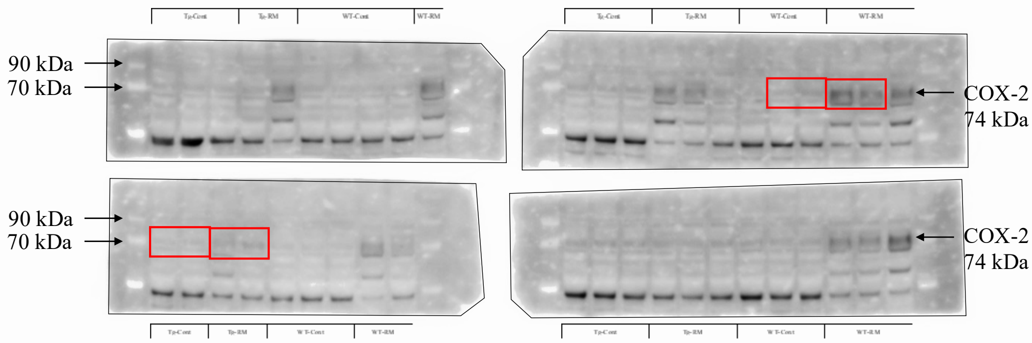

L. α-Tubulin for K from the same membrane

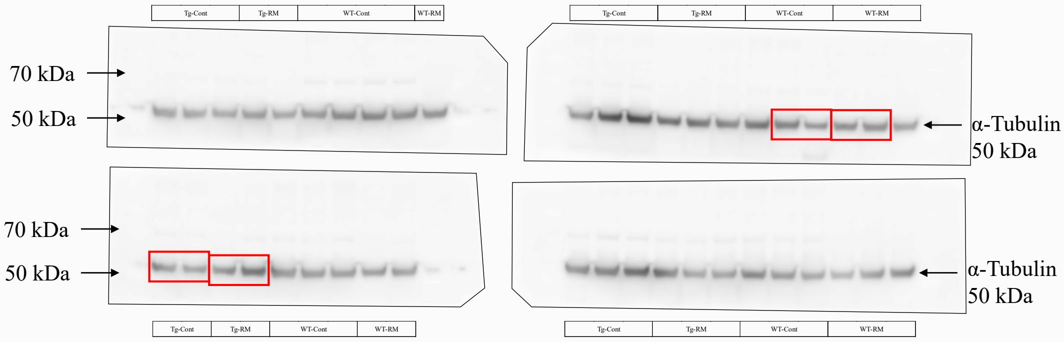

M. GPX4

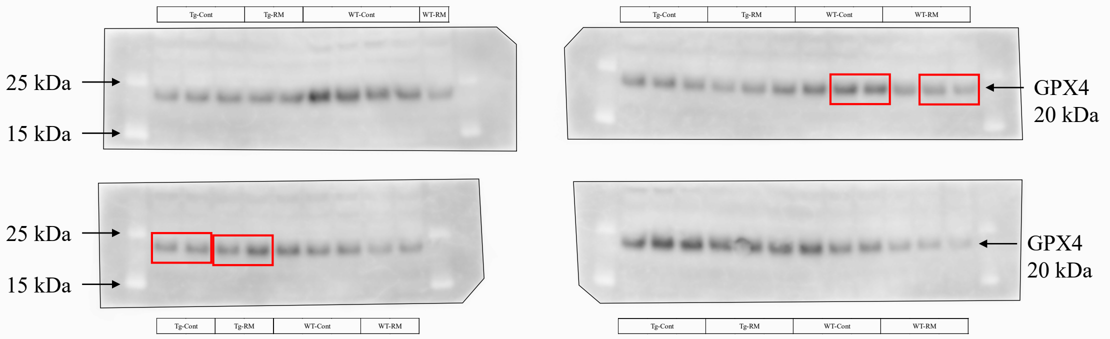

N. α-Tubulin for M from the same membrane

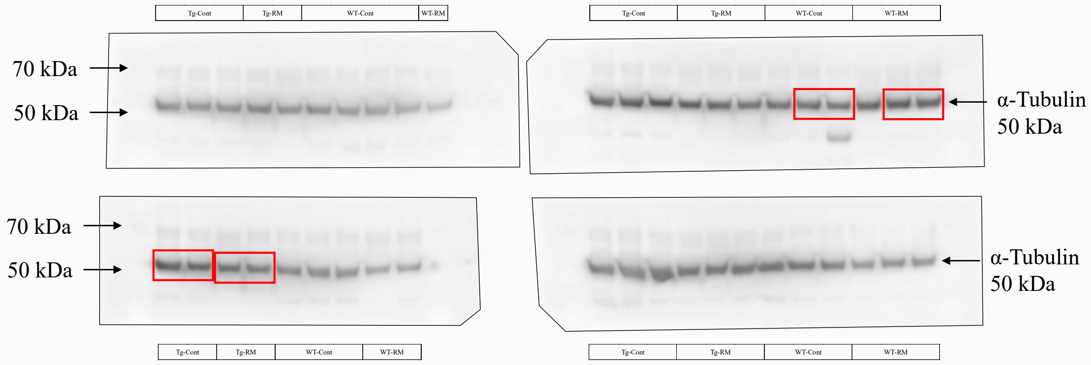

Western blotting of ACSL4, SLC7A11, COX-2, GPX4 and α-Tubulin on day 1 (A to G) and day 3 (H to N). Black lines represent the edge of each cut membrane. Red boxes show the regions of the original blots used in main figures.
